# New insights into molecular pathways associated with flatfish ovarian development and atresia revealed by transcriptional analysis

**DOI:** 10.1186/1471-2164-10-434

**Published:** 2009-09-15

**Authors:** Angèle Tingaud-Sequeira, François Chauvigné, Juanjo Lozano, María J Agulleiro, Esther Asensio, Joan Cerdà

**Affiliations:** 1 Laboratory of Institut de Recerca i Tecnologia Agroalimentàries (IRTA)-Institut de Ciències del Mar, Consejo Superior de Investigaciones Científicas (CSIC), 08003 Barcelona, Spain; 2 Génomique et Physiologie des Poissons, Université Bordeaux 1, UMR NuAGe, 33405 Talence, France; 3 Centro de Investigación Biomédica en Red de Enfermedades Hepáticas y Digestivas (CIBERehd), 08036 Barcelona, Spain; 4 Instituto de Acuicultura Torre de la Sal (CSIC), 12595 Castellón, Spain; 5 IFAPA Centro El Toruño, Junta de Andalucía, Cádiz, Spain

## Abstract

**Background:**

The Senegalese sole (*Solea senegalensis*) is a marine flatfish of increasing commercial interest. However, the reproduction of this species in captivity is not yet controlled mainly because of the poor knowledge on its reproductive physiology, as it occurs for other non-salmonid marine teleosts that exhibit group-synchronous ovarian follicle development. In order to investigate intra-ovarian molecular mechanisms in Senegalese sole, the aim of the present study was to identify differentially expressed genes in the ovary during oocyte growth (vitellogenesis), maturation and ovarian follicle atresia using a recently developed oligonucleotide microarray.

**Results:**

Microarray analysis led to the identification of 118 differentially expressed transcripts, of which 20 and 8 were monitored by real-time PCR and in situ hybridization, respectively. During vitellogenesis, many up-regulated ovarian transcripts had putative mitochondrial function/location suggesting high energy production (NADH dehydrogenase subunits, cytochromes) and increased antioxidant protection (selenoprotein W2a), whereas other regulated transcripts were related to cytoskeleton and zona radiata organization (zona glycoprotein 3, alpha and beta actin, keratin 8), intracellular signalling pathways (heat shock protein 90, Ras homolog member G), cell-to-cell and cell-to-matrix interactions (beta 1 integrin, thrombospondin 4b), and the maternal RNA pool (transducer of ERBB2 1a, neurexin 1a). Transcripts up-regulated in the ovary during oocyte maturation included ion transporters (Na^+^-K^+^-ATPase subunits), probably required for oocyte hydration, as well as a proteinase inhibitor (alpha-2-macroglobulin) and a vesicle calcium sensor protein (extended synaptotagmin-2-A). During follicular atresia, few transcripts were found to be up-regulated, but remarkably most of them were localized in follicular cells of atretic follicles, and they had inferred roles in lipid transport (apolipoprotein C-I), chemotaxis (leukocyte cell-derived chemotaxin 2,), angiogenesis (thrombospondin), and prevention of apoptosis (S100a10 calcium binding protein).

**Conclusion:**

This study has identified a number of differentially expressed genes in the ovary that were not previously found to be regulated during ovarian development in marine fish. Specifically, we found evidence, for the first time in teleosts, of the activation of chemoattractant, angiogenic and antiapoptotic pathways in hypertrophied follicular cells at the onset of ovarian atresia.

## Background

Our understanding of the molecular pathways underlying reproductive processes and oogenesis in vertebrates is still limited even in mammalian models. Teleosts show the most diversified reproductive strategies among vertebrates making it even more difficult to uncover the underlying molecular mechanisms. In addition, several factors can modulate the reproductive processes of teleost fish such as photoperiod and temperature [1,2], nutrition [3], captivity [4] and endocrine disruptors [5,6]. Accordingly, most studies to date on female teleosts have mainly investigated the effect of these conditions on the circulating sex hormone levels or the reproductive success in terms of spawning performance (e.g., fecundity, egg and larval survival). However, the molecular and cellular mechanisms involved still remain poorly understood.

The development of methods for large-scale gene expression analysis (e.g., microarrays) in model fish species, such as the zebrafish (*Danio rerio*), as well as in salmonids is improving our knowledge of the molecular basis of ovarian physiology in teleosts [7]. In the zebrafish, cDNA- and oligo-based microarrays have been employed to assess the transcriptome profile of differentiating and adult gonads. These studies have identified a number of genes involved in mitochondrial organization and biogenesis, cell growth and maintenance, and germ-line differentiation, as well as some with sexually dimorphic co-expression in both the gonads and the brains [8-10]. Mass sequencing of zebrafish expressed sequence tags (ESTs) from the ovary [11], or from isolated fully-grown ovarian follicles through serial analysis of gene expression (SAGE) [12], has also discovered germ cell-specific genes and established the complete sequence data set of maternal mRNA stored in oocytes at the end of oogenesis. In rainbow trout (*Oncorhynchus mykiss*) and coho salmon (*O. kisutch*), cDNA microarrays printed on slides or nylon membranes, as well as reciprocal suppression subtractive hybridization (SSH), were used to investigate changes in the ovarian transcriptome during primary growth and maturation and bacterial lipopolysaccharide-induced ovarian apoptosis [13-17]. These studies revealed changes in the expression of genes involved in cellular organization and extracellular matrix (ECM) remodelling, immunoregulation, apoptosis, cell cycling, and in different endocrine and paracrine systems, which might be important during ovarian development. The control of ovulation by either hormonal induction or photoperiod manipulation has also been shown to induce differences in the egg mRNA abundance of specific genes, which may affect their developmental competence [18].

However, despite the significant information obtained from zebrafish and salmonids, no data is currently available on the changes of the transcriptome during ovarian development in other marine teleosts, such as flatfish, some of which are of economical importance. Particularly, little is known on the molecular pathways involved in ovarian follicular atresia, a degenerative and resorptive process of ovarian follicles that determines fecundity in both natural and captive conditions [19-22]. The use of functional genomics approaches would contribute with the identification of molecular signatures associated with abnormal ovarian development or premature ovarian regression in cultured fish species, thus providing potentially useful markers to control sexual maturation.

The Senegalese sole, *Solea senegalensis*, is a marine flatfish of high commercial value in Southern Europe and Asia [23]. However, the industrial production of this species is largely based on wild breeders after long periods of acclimation to captivity, and reproduction is not yet controlled [26]. The F1 generation of fish raised in captivity often fail to reproduce naturally because egg fertilization is dramatically reduced [24,25]. In some F1 females, an increased ovarian follicle atresia and/or dysfunctions of the ovulatory process might also occur, but no precise studies have been performed to clarify this phenomenon. In order to obtain information on the molecular basis of ovarian development in Senegalese sole, the present study aimed at performing a transcriptomic analysis of the ovary during oocyte growth (vitellogenesis), maturation and follicular atresia using a recently developed oligonucleotide microarray [27]. The analysis revealed the differential expression of more than one hundred genes during ovarian development, some of them with yet unknown functions in the fish ovary. In addition, determination of the cell type-specific expression in the ovary of selected transcripts suggest the activation of genes presumably involved in chemotaxis, angiogenesis and prevention of apoptosis in follicular cells of atretic follicles, which have not been described before in teleosts during ovarian atresia.

## Results

### Stages of ovarian development

**Figure 1:**
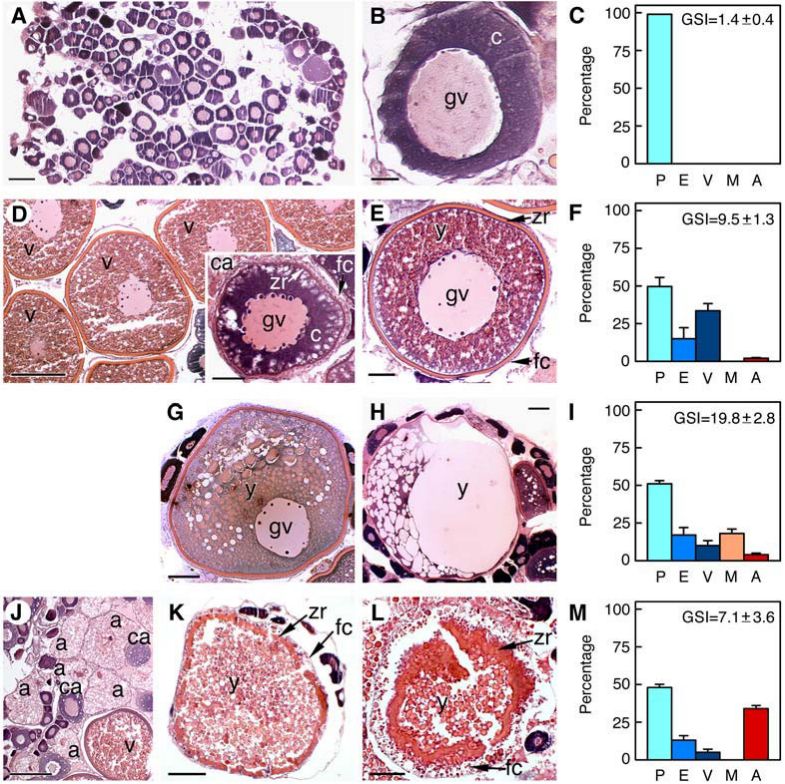
**Developmental stage of the Senegalese sole ovaries used for microarray analysis**. Representative light micrographs of histological sections of the ovary (*n *= 3 females) stained with hematoxylin-eosin (A, B, D, E, G, H, and J-L), and frequency of ovarian follicles in the ovary (C, F, I and M) at each ovarian developmental stage, previtellogenic (A and B), vitellogenic (D and E), mature (G and H) and atretic (J-L). Data on the frequency of ovarian follicles are the mean ± SEM (*n *= 3 females). (B) Oocyte at primary growth stage. (D inset) Ovarian follicle containing a cortical alveolus stage oocyte. (G) Follicle at early maturation (note the migration of the germinal vesicle). (H) Follicle containing a mature oocyte (the germinal vesicle is not observed and yolk is fused). (K) Ovarian follicle at early atresia. (L) Follicle at advanced atresia. gv, germinal vesicle; c, oocyte cytoplasm; y, yolk; zr, zona radiata; fc, follicular cells; ca, cortical alveolus stage oocyte. P, previtellogenic follicle; E, early vitellogenic follicle; V, vitellogenic follicle; M, mature follicle; A, atretic follicle. Bars, 200 μm (D, J), 100 μm (A, G, H, K), 50 μm (E, D inset, L), 20 μm (B).

To identify differentially expressed genes during ovarian development in Senegalese sole, samples of ovarian tissue were collected from adult females sacrificed throughout the annual reproductive cycle [28,29] or after hormonal treatment (Figure 1). Thus, samples of ovaries at previtellogenesis (Figure 1A and 1B), vitellogenesis (Figure 1D and 1E), maturation (Figure 1G and 1H), and undergoing follicular atresia (Figure 1J, K and 1L), were used for transcriptome analysis. As many other fractional spawner teleosts, the Senegalese sole has a group-synchronous ovary in which follicles of all sizes up through vitellogenesis are present at any time, and populations (or clutches) of follicles are periodically recruited into maturation from a population of oocytes in late vitellogenic stages [30]. Therefore, the increased frequency of vitellogenic, mature or atretic ovarian follicles in the ovary, as determined by histological analysis (Figure 1C, F, I and 1M), defined the ovarian developmental stages used in the present study.

The previtellogenic ovary was formed by exclusively ovarian follicles with oocytes at the primary growth stage (oocyte/follicle diameter up to approximately 150 μm) in which vitellogenin incorporation and yolk formation did not yet start (Figure 1A-1C). In the vitellogenic ovary, a population of follicles were recruited into vitellogenesis, and consequently the proportion of follicles at the primary growth stage decreased (Figure 1D-1F). At this stage, follicles containing oocytes at the cortical alveolus stage (up to approximately 300 μm), characterized by the presence of nascent cortical alveoli within the ooplasm, were more abundant (Figure 1D, inset). Vitellogenic oocytes surrounded by the zona radiata and the somatic follicular cells, granulosa and theca cells, increased in size (up to 500 μm in diameter at late vitellogenesis) and their cytoplasm was filled with yolk granules where vitellogenin-derived yolk proteins are stored (Figure 1E). As a result of this growing phase, the gonadosomatic index (GSI) of females increased by approximately 7-fold (Figure 1C and 1F).

Maturing ovaries containing follicle-enclosed oocytes undergoing meiosis resumption, and ovaries carrying mature oocytes prior to ovulation, were collected 24-48 h after treatment of vitellogenic females with gonadotropin-releasing hormone agonist [D-Ala^6^, Pro^9^, NEt] (GnRHa) [24]. In the mature ovary, a population of follicle-enclosed oocytes at late stages of vitellogenesis was further recruited into maturation (Figure 1G-1I). In these oocytes, the germinal vesicle migrates towards the animal pole and yolk globules fuse one another (Figure 1G), eventually forming a large mass of yolk (Figure 1H). The mature oocyte reached 800-900 μm in diameter due to water uptake (hydration), resulting in a further 2-fold increase of the GSI (Figure 1F and 1I).

**Figure 2:**
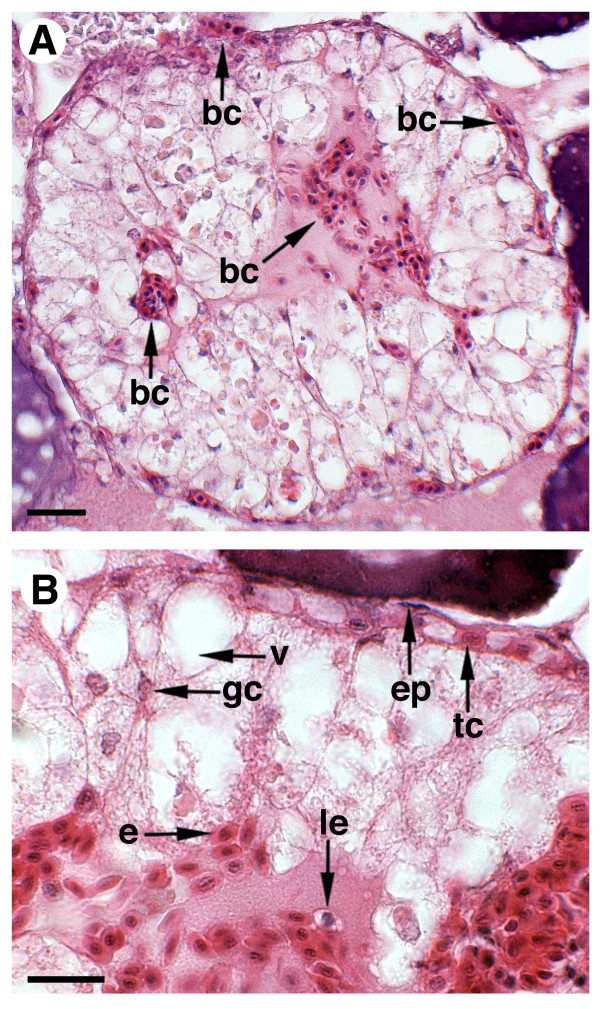
**Photomicrographs of ovarian follicles at advanced atresia**. Light micrographs of histological sections stained with hematoxylin-eosin. bc, blood cells; ep, epithelium; tc, theca cells; v, vacuole; gc, granulosa cells; e, erythrocytes; le, leukocytes. Bars, 20 μm.

Finally, atretic ovaries were collected from females showing spontaneously occurring ovarian follicle atresia during the spawning season, or induced after GnRHa treatment. In these ovaries, approximately up to 30-40% of the ovarian follicles showed different levels of atresia and maturing/mature oocytes were absent (Figure 1J-1M). In early atretic follicles, vitellogenic oocytes shrank, the zona radiata folded, and follicles became irregularly shaped (Figure 1K and 1L). The follicular cells were hypertrophied and the theca was poorly developed. Advanced follicular atresia was characterized by breakdown and resorption of the zona radiata, and the appearance of highly columnar follicular cells apparently showing an intense phagocytic activity as suggested by the presence of large vacuoles (Figure 2). At this stage, accumulation of blood cells, erythrocytes and leukocytes in the follicle, as well as in the oocyte, was also noted (Figure 2B).

### Microarray analysis

Differential gene expression in the four ovarian developmental stages was determined using a Senegalese sole-specific oligonucleotide microarray containing 60-mer probes representing 5,087 unique genes [27]. This platform was previously designed from a Senegalese sole EST database derived from a multi-tissue normalized cDNA library from different adult tissues (including ovaries at different developmental stage) and larval and juvenile stages [27]. Therefore, although this platform was not ovary-specific and most likely did not contain all the transcripts expressed in the sole ovary, its was useful to obtain a first insight into the overall changes of gene expression during ovarian development.

To determine the false discovery rate (FDR) in each of the differential gene expression experiments (vitellogenic *vs*. previtellogenic ovaries, mature *vs*. vitellogenic ovaries, and atretic *vs*. vitellogenic/mature ovaries), an additional microarray experiment was performed by hybridizing differentially labelled (Cy3 and Cy5) aliquots of amplified RNA (aRNA) from the same sample (previtellogenic ovary). As expected, there were few differences between the Cy3 and Cy5 signals for most of the microarray spots in these experiments giving an estimated overall FDR of 3.0, 3.8 and 5.6% for vitellogenic *vs*. previtellogenic ovaries, mature *vs*. vitellogenic ovaries, and atretic *vs*. vitellogenic/mature ovaries, respectively (see Additional file 1).

Microarray data analysis indicated significant (*p *< 0.01) regulation of genes in vitellogenic (46 ESTs), mature (46 ESTs) and atretic ovaries (26 ESTs), which showed fold change (FC) values from 1.4 up to 5.1. These ESTs, and the corresponding GenBank accession numbers are listed in Tables 1, 2 and 3. In Table 3, differential expressed genes in atretic ovaries relative to vitellogenic or mature ovaries are pooled together. Some of these ESTs (26%) could not be annotated, even after sequencing the respective clones from the 5' end, and are not included in these tables.

**Table 1:** Transcripts regulated in vitellogenic ovary relative to previtellogenic ovary

**Clone ID**	**GenBank accession**	**FC^a^**	**UniProtKB/TrEMBL entry**	**Swiss-Prot hit**	**BLAST** **E-value**	**Length (% identity)^b^**	**Gene Symbol**
pgsP0015N21	FF286629	+5.13	Q3ZLC7	Selenoprotein W2a [*Oreochromis mossambicus*]	4E-04	27 (70%)	*sepw2a*
pgsP0003P21	FF282633	+4.64	JC1348	Hypothetical 18 K protein, mitochondrion [*Carassius auratus*]	2E-06	40 (70%)	
pgsP0005D08	FF283023	+4.49		Unknown			
pgsP0017G22	FF287169	+3.94	Q0KJ14	Cytochrome c oxidase subunit I [*Solea senegalensis*]	3E-84	230 (92%)	*cox1*
pgsP0029L14	FF291468	+3.67	Q7ZUR6	Muscle-specific beta 1 integrin binding protein 2 [*Danio rerio*]	2E-83	192 (78%)	*mibp2*
pgsP0018D11	FF287436	+3.56	Q75v54	Cytochrome b [*S. senegalensis*]	8E-74	165 (93%)	*cytb*
pgsP0019B22	FF287743	+3.34	A8R7E8	Cytosolic heat shock protein 90 beta [*S. senegalensis*]	6E-70	133 (100%)	*hsp90b*
pgsP0008A11	FF283945	+3.21	Q0KJ09	NADH dehydrogenase subunit 3 [*S. senegalensis*]	1E-43	111 (84%)	*nd3*
pgsP0003A24	FF282359	+3.00	B1B560	Beta actin isoform 1 [*S. senegalensis*]	4E-22	51 (100%)	*bactin1*
pgsP0020D03	FF288118	+2.93	P79893	Chorion protein (zona protein 3) [*Sparus aurata*]	8E-69	160 (70%)	*zp3*
pgsP0021P08	FF288738	+2.69	Q0KJ16	NADH dehydrogenase subunit 1 [*S. senegalensis*]	2E-124	280 (88%)	*nd1*
pgsP0013B15	FF285673	+2.62	Q7SZR1	Transducer of ERBB2, 1a [*Danio rerio*]	1E-57	175 (77%)	*tob1a*
pgsP0007G02	FF283724	+2.59	Q6IQR3	Alpha actin [*D. rerio*]	7E-123	282 (83%)	*actc1l*
pgsP0025N01	FF290105	+2.47	Q805D1	Tropomyosin1-1 [*Takifugu rubripes*]	1E-19	55 (90%)	*tpm1-1*
pgsP0029A20	FF291229	+2.41	Q0P4B4	Transgelin [*D. rerio*]	4E-60	141 (79%)	*tagln*
pgsP0028P05	FF291191	+2.29	Q6PFN7	Protein arginine methyltransferase 1 [*D. rerio*]	1E-167	308 (95%)	*prmt1*
pgsP0015D24	FF286403	+2.12		Unknown			
pgsP0020O05	FF288363	+2.11		Unknown			
pgsP0014A08	FF285984	+2.09		Unknown			
pgsP0029C16	FF291271	+1.99	A1XQX0	Neurexin 1a [*D. rerio*]	3E-132	266 (84%)	*nrxn1a*
pgsP0015H15	FF286488	+1.98		Unknown			
pgsP0030I24	FF291759	+1.96		Unknown			
pgsP0029L19	FF291472	+1.92	Q8JGW0	Thrombospondin 4b [*D. rerio*]	1E-100	175 (97%)	*thbs4b*
pgsP0004D01	FF282692	+1.93	Q5ZMG8	Ras homolog member G (rho G) [*Gallus gallus*]	2E-90	162 (93%)	*rhog*
pgsP0017K07	FF287242	+1.90	Q6NWF6	Keratin, type II cytoskeletal 8 [*D. rerio*]	4E-33	79 (94%)	*krt8*
pgsP0028F14	FF290985	+1.89	Q9UMS5	Putative homeodomain transcription factor 1 [*Homo sapiens*]	4E-56	144 (70%)	*phtf1*
pgsP0016G07	FF286813	+1.88		Unknown			
pgsP0009A17	FF284280	+1.87	Q2YDR3	Inositol monophosphatase 3 [*D. rerio*]	6E-13	74 (84%)	*impa3*
pgsP0027D14	FF290593	+1.85	Q8AY63	Creatine kinase, brain [*D. rerio*]	7E-84	179 (90%)	*ckb*
pgsP0020F22	FF288178	+1.82	Q4U0S2	Smooth muscle myosin heavy chain [*D. rerio*]	1E-97	180 (87%)	*myh11*
pgsP0027C08	FF290565	+1.80	Q3T0R7	3-Ketoacyl-CoA thiolase, mitochondrial [*Bos taurus*]	1E-30	76 (82%)	*acaa2*
pgsP0028D08	FF290934	+1.76		Unknown			
pgsP0008G08	FF284066	+1.74		Unknown			
pgsP0001G21	FF281897	+1.74	A3KQ53	Novel protein similar to vertebrate serum/glucocorticoid regulated kinase (SGK) [*D. rerio*]	2E-45	90 (81%)	*sgk*
pgsP0013L08	FF285880	-5.32	Q1L8U4	Novel protein similar to vertebrate CDC-like kinase 2 (CLK2) [*D. rerio*]	4E-91	184 (87%)	*si:ch211-81a5.7*
pgsP0028D18	FF290943	-4.80	Q6PHK4	Alanine-glyoxylate aminotransferase [*D. rerio*]	2E-64	150 (76%)	*agxt*
pgsP0020C06	FF288101	-3.58		Unknown			
pgsP0028H11	FF291026	-3.17	Q7T376	Novel protein similar to vertebrate CD53 molecule [*D. rerio*]	8E-24	92 (55%)	*zgc:64051*
pgsP0027F18	FF290638	-2.23		Unknown			
pgsP0028J05	FF291062	-2.22	Q8HXG6	NADH dehydrogenase [ubiquinone] 1 alpha subcomplex subunit 11 [*B. taurus*]	2E-23	133 (44%)	*ndufa11*
pgsP0008I12	FF284114	-2.12	Q6NY77	Dihydrodipicolinate synthase-like, mitochondrial [*D. rerio*]	2E-66	172 (77%)	*zgc:77082*
pgsP0007P01	FF283914	-2.12	Q7TQ08	Scinderin [*Rattus norvegicus*]	3E-79	193 (70%)	*scin*
pgsP0009H07	FF284426	-2.10	Q2TAT1	Hypothetical protein LOC735233 [*Xenopus laevis*]	1E-24	166 (43%)	*MGC130935*
pgsP0016E15	FF286777	-2.06	Q8UW64	Proteasome subunit beta type-9 [*Oryzias latipes*]	3E-41	98 (84%)	*psmb9a*
pgsP0004A14	FF282647	-2.01	Q5JBI1	Carboxypeptidase H [*Paralichthys olivaceus*]	4E-58	133 (94%)	*cph*
pgsP0012M12	FF285564	-1.89	Q6ZM96	Coiled-coil domain containing 90B [*D. rerio*]	1E-22	107 (61%)	*ccdc90b*

^a^Fold change; ^b ^Extent of BLASTX hit aligned region (in amino acids), and percent identity over the aligned region.

**Table 2:** Transcripts regulated in mature ovary relative to vitellogenic ovary

**Clone ID**	**GenBank accession**	**FC^a^**	**UniProtKB/TrEMBL entry**	**Swiss-Prot hit**	**BLAST** **E-value^b^**	**Length (% identity)**	**Gene Symbol**
pgsP0012B12	FF285326	+4.54	B6NTA9	Hypothetical protein BRAFLDRAFT_128798 [*Branchiostoma floridae*]	2E-14	107 (41%)	*BRAFLDRAFT_128798*
pgsP0016M08	FF286940	+2.56	Q6NYH5	ATPase, Na+\/K+ transporting, beta 1a polypeptide [*Danio rerio*]	2E-10	39 (76%)	*atp1b1a*
pgsP0013H16	FF285803	+2.51	Q8AY58	Sodium/potassium ATPase alpha subunit isoform 1 [*Fundulus heteroclitus*]	2E-118	213 (95%)	*atp1a1*
pgsP0017B15	FF287055	+2.22	Q67EX5	Alpha-2-macroglobulin [*Sparus aurata*]	4E-81	221 (70%)	*a*2*m*
pgsP0023I10	FF289288	+1.97	Q56V59	Sodium potassium ATPase beta subunit [*Rhabdosargus sarba*]	1E-79	216 (62%)	*atpb*
pgsP0030L16	FF291822	+1.86	Q694W8	Myosin 10 [*Xenopus laevis*]	5E-32	78 (83%)	*myo10*
pgsP0027A07	FF290524	+1.75	Q9UL19	Retinoic acid receptor responder protein 3 [*Homo sapiens*]	5E-15	108 (40%)	*rarres3*
pgsP0007C11	FF283652	+1.69		Unknown			
pgsP0009C03	FF284311	+1.64		Unknown			
pgsP0022M06	FF289024	+1.64	Q7ZWB6	Thioredoxin interacting protein [*D. rerio*]	2E-108	296 (68%)	*txnip*
pgsP0013B15	FF285673	+1.61	Q7SZR1	Transducer of ERBB2, 1a [*D. rerio*]	1E-57	175 (77%)	*tob1a*
pgsP0022B09	FF288784	+1.60	A9ZTB5	Ribosomal protein L36 [*Solea senegalensis*]	3E-46	94 (100%)	*rpl36*
pgsP0003B14	FF282371	+1.57		Unknown			
pgsP0027G11	FF290655	+1.55		Unknown			
pgsP0019D13	FF287779	+1.45		Unknown			
pgsP0005H14	FF283112	+1.42		Unknown			
pgsP0001N17	FF282035	+1.41	Q5FWL4	Extended synaptotagmin-2-A [*X. laevis*]	7E-58	144 (74%)	*e-syt2-a*
pgsP0022B24	FF288799	+1.40	Q5NU14	Makorin RING zinc finger protein 1a [*Takifugu rubripes*]	4E-64	224 (50%)	*mkrn1*
pgsP0022J20	FF288970	+1.40	Q9WTY9	Mitogen-activated protein kinase p38delta [*Rattus norvegicus*]	3E-29	127 (49%)	*mapk13*
pgsP0027B20	FF290555	+1.39	Q6P5M5	Novel protein similar to vertebrate ADP-ribosylation factor 4 [*D. rerio*]	6E-76	174 (86%)	*zgc:77650*
pgsP0021P08	FF288738	+1.39	Q0KJ16	NADH dehydrogenase subunit 1 [*S. senegalensis*]	2E-124	280 (88%)	*nd1*
pgsP0017G22	FF287169	+1.38	Q0KJ14	Cytochrome c oxidase subunit I [*S. senegalensis*]	3E-84	230 (92%)	*cox1*
pgsP0007O02	FF283892	+1.37	A8WGP7	UDP-glucose dehydrogenase [*D. rerio*]	1E-38	104 (75%)	*ugdh*
pgsP0018P15	FF287693	+1.36	Q63ZU3	Myeloid-associated differentiation marker homolog [*X. laevis*]	3E-15	93 (47%)	*myadm*
pgsP0029B12	FF291245	+1.36	Q4QY72	Type II keratin E3-like protein [*S. aurata*]	4E-108	217 (92%)	
pgsP0006O02	FF283575	+1.32		Unknown			
pgsP0027D07	FF290586	-2.49		Unknown			
pgsP0017L02	FF287257	-2.31	Q9PTQ8	Type II Na/Pi cotransport system protein [*D. rerio*]	7E-32	128 (73%)	*slc34a2a*
pgsP0023F13	FF289226	-2.11	Q5M901	Myosin binding protein H [*X. tropicalis*]	1E-46	139 (64%)	*mybph*
pgsP0002P23	FF282343	-1.80		Unknown			
pgsP0019F07	FF287815	-1.72	Q5XWB3	Ubiquitous gelsolin [*D. rerio*]	2E-81	192 (72%)	*gsna*
pgsP0009J15	FF284472	-1.71	Q007T0	Succinate dehydrogenase [ubiquinone] iron-sulfur subunit, mitochondrial [*Sus scrofa*]	1E-08	30 (93%)	*sdhb*
pgsP0023H12	FF289266	-1.71	A2CEC9	Novel protein similar to centaurin, delta 2 [*D. rerio*]	5E-36	272 (36%)	*LOC100005008*
pgsP0009A17	FF284280	-1.70	Q28CL4	Inositol monophosphatase 3 [*X. tropicalis*]	7E-12	66 (71%)	*impa3*
pgsP0024D08	FF289528	-1.59		Unknown			
pgsP0022O08	FF289072	-1.53	Q7SXH8	Coagulation factor II (thrombin) [*D. rerio*]	4E-88	140 (76%)	*f*2
pgsP0010A15	FF284617	-1.48	Q6J514	Monocytic leukemia zinc finger protein [*D. rerio*]	3E-22	96 (90%)	*myst3*
pgsP0029E16	FF291319	-1.46	Q6P8F7	Fructose-1,6-bisphosphatase [*X. tropicalis*]	1E-132	231 (77%)	*fbp1*
pgsP0019H08	FF287862	-1.43	XP_001920639	Similar to apolipoprotein L, 3 [*D. rerio*]	9E-60	115 (72%)	*LOC100150119*
pgsP0015B20	FF286358	-1.43	Q8AYL1	Zona pellucida C2 [*Oryzias latipes*]	9E-69	161 (67%)	*zpc2*
pgsP0018J23	FF287569	-1.40	B2R4S9	Histone 1, H2bc, isoform CRA_a [*H. sapiens*]	3E-35	77 (97%)	*hist1h2be*
pgsP0029D07	FF291286	-1.39	P62260	14-3-3 protein epsilon [*R. norvegicus*]	2E-20	65 (76%)	*ywhae*
pgsP0030I24	FF291759	-1.39		Unknown			
pgsP0013O20	FF285955	-1.38		Unknown			
pgsP0020J11	FF288260	-1.36		Unknown			
pgsP0023L15	FF289356	-1.33		Unknown			

^a^Fold change; ^b ^Extent of BLASTX hit aligned region (in amino acids), and percent identity over the aligned region.

**Table 3:** Transcripts regulated in atretic ovary relative to vitellogenic/mature ovary

**Clone ID**	**GenBank accession**	**FC^a^**	**UniProtKB/TrEMBL entry**	**Swiss-Prot hit**	**BLAST** **E-value^b^**	**Length (% identity)**	**Gene Symbol**
pgsP0015C05	FF286365	+5.13	B6DUH2	Apolipoprotein C-I [*Hemibarbus mylodon*]	1E-08	64 (46%)	*apoc1*
pgsP0002L08	FF282262	+3.36		Unknown			
pgsP0007K07	FF283810	+2.90	A8D3J2	Leukocyte cell-derived chemotaxin 2 [*Lates calcarifer*]	3E-53	129 (77%)	*lect2*
pgsP0010O12	FF284909	+2.35	Q28178	Thrombospondin [*Bos taurus*]	1E-27	76 (72%)	*thbs*
pgsP0022K21	FF288994	+2.05	B5X719	Heme-binding protein 2 [*Salmo salar*]	2E-32	85 (40%)	*hebp2*
pgsP0008C17	FF283994	+1.93	Q5KSU5	Apolipoprotein A-I [*Takifugu rubripes*]	7E-53	197 (49%)	*apoa1*
pgsP0020M08	FF288324	+1.84	A8HG28	S100-like calcium binding protein [*Epinephelus coioides*]	6E-38	101 (74%)	*s*100
pgsP0005K16	FF283180	+1.62	Q6TH14	Enolase [*Danio rerio*]	1E-91	190 (92%)	*eno3*
pgsP0019F17	FF287823	+1.62		Unknown			
pgsP0013O20	FF285955	+1.49		Unknown			
pgsP0012B12	FF285326	-4.01	B6NTA9	Hypothetical protein BRAFLDRAFT_128798 [*Branchiostoma floridae*]	2E-14	107 (41%)	*BRAFLDRAFT_128798*
pgsP0017B15	FF287055	-2.35	Q67EX5	Alpha-2-macroglobulin [*Sparus aurata*]	4E-81	221 (70%)	*a*2*m*
pgsP0025P21	FF290169	-2.08	XP_001519899	Similar to hCG2006161, partial [*Ornithorhynchus anatinus*]	4E-04	54 (51%)	*LOC100090881*
pgsP0006E10	FF283378	-1.93	Q66KL2	C-terminal binding protein 1 [*Xenopus tropicalis*]	1E-61	178 (78%)	*ctbp1*
pgsP0013I09	FF285819	-1.88		Unknown			
pgsP0021L12	FF288651	-1.83	Q4H447	Elongation factor 1 alpha [*Hyla japonica*]	0.64	16 (69%)	*ef1a*
pgsP0002C23	FF282126	-1.74	Q90YM8	Cytoplasmic FMR1 interacting protein 1 homolog [*D. rerio*]	9E-119	227 (91%)	*cyfip1*
pgsP0013H18	FF285805	-1.68	XP_001340467.2	wu:fb21f05 [*D. rerio*]	2E-23	112 (50%)	*wu:fb21f05*
pgsP0024C11	FF289508	-1.68		Unknown			
pgsP0007E08	FF283689	-1.58	B0UY57	Novel protein similar to vertebrate RAP1 interacting factor homolog [*D. rerio*]	7E-38	112 (76%)	*rif1*
pgsP0009C03	FF284311	-1.58		Unknown			
pgsP0013B03	FF285662	-1.58		Unknown			
pgsP0014A17	FF285991	-1.56	B5X408	Elongation of very long chain fatty acids protein 1 [*S. salar*]	1E-24	60 (60%)	*elovl1b*
pgsP0002P23	FF282343	-1.56	B5X202	Zinc finger protein 576 [*S. salar*]	1E-04	65 (41%)	*znf576*
pgsP0006D18	FF283364	-1.55	B5X308	Golgi membrane protein 1 [*S. salar*]	2E-19	55 (60%)	*golm1*
pgsP0030G11	FF291702	-1.55		Unknown			

^a^Fold change; ^b ^Extent of BLASTX hit aligned region (in amino acids), and percent identity over the aligned region.

### Gene ontology annotation

To obtain a first assessment of the more important physiological processes occurring during ovarian development, gene ontology (GO) analysis was carried out using the BLAST2GO v1 program [31]. Most of the annotated ESTs (93%) had GO assignments, and many of those had 3-6 assignments each (49%) and a significant proportion (34%) had 7 or more assignments.

**Figure 3:**
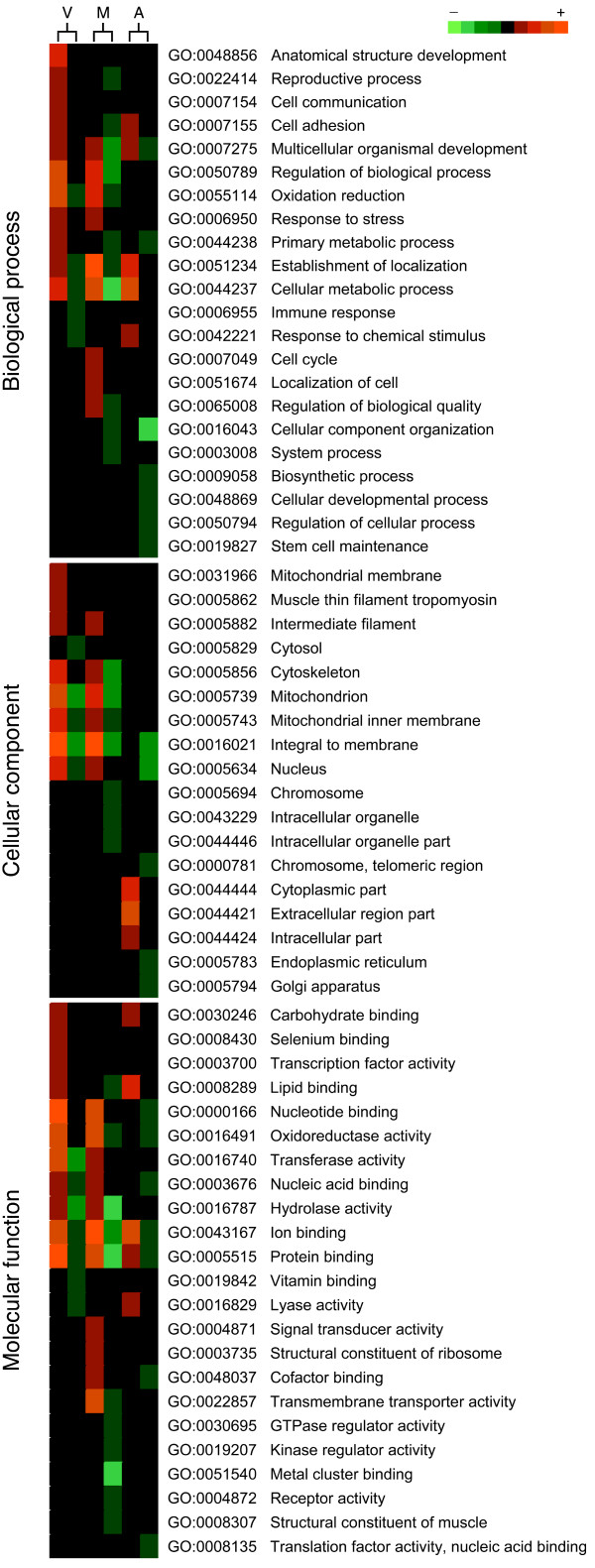
**Gene ontology (GO) analysis of differentially expressed genes in the Senegalese sole ovary**. Genes regulated during vitellogenesis (V), maturation (M) and atresia (A) were classified according to GO terms biological process (level 3), cellular component (level 5) and molecular function (level 3). For each GO term, the number of differentially expressed transcripts detected in the microarray is indicated using a color intensity scale. Red and green are used for over and under abundance, respectively, whereas black indicates no change.

Figure 3 shows the differentially expressed genes in the three ovarian stages (vitellogenesis, maturation and follicular atresia) classified according to GO terms biological process (level 3), cellular component (level 5) and molecular function (level 3). During vitellogenesis, the majority of regulated ESTs were dedicated to metabolic process, oxidation reduction, regulation and anatomical structure development, in the biological process category. A similar distribution of GO terms was seen within the EST cluster regulated during maturation, although in this case transcripts related to cell cycle, localization of cell, cellular component organization, and system process, were also detected. During ovarian follicle atresia, most regulated genes fall in the cellular metabolism, establishment of localization, and cellular component organization attributes.

During vitellogenesis and maturation, most protein products were mainly inferred to be associated with mitochondria based on the cellular component category, although some also might be in the cytoskeleton (specially during vitellogenesis), the nucleus, and intracellularly in organelles. Interestingly, during atresia, the products of most of the up-regulated genes showed putative extracellular location, whereas the products of the down-regulated genes had membrane and nucleus locations.

Finally, classification using the molecular function category indicated that most of the gene products regulated during vitellogenesis and maturation were dedicated to binding and catalytic functions, including nucleotide binding, protein binding, ion binding, and transferase and hydrolase activities. However, products involved in transmembrane transporter activity only appeared during maturation. In the atretic ovary, the majority of products were associated with ion and lipid binding.

### Vitellogenesis

In the vitellogenic ovary, 34 and 12 transcripts were found to be up- and down-regulated, respectively, relative to previtellogenic ovaries; 35 had a significant hit in Swiss-Prot database (Table 1). The most highly up-regulated transcripts corresponded to selenoprotein W2a (*sepw2a*), hypothetical 18K protein from *Carassius auratus *mitochondrion, muscle-specific beta 1 integrin binding protein 2 (*mibp2*), zona pellucida protein 3 (*zp3*), cytochrome c oxidase subunit I (*cox1*), cytochrome b (*cytb*), cytosolic heat shock protein 90 beta (*hsp90b*), NADH dehydrogenase subunit 3 (*nd3*) and 1 (*nd1*), and beta actin 1 (*bactin1*). The sequence similarity of clone pgsP0015N21 to tilapia (*Oreochromis mossambicus*) *sepw2a *was low (4E-04) possibly because its nucleotide sequence only covered the C terminus of tilapia *sepw2a*.

Other up-regulated genes in the vitellogenic ovary, but at lower levels, were additional components of the cytoskeleton, such as alpha actin (*actc1l*), keratin 8 (*krt8*), tropomyosin1-1 (*tpm1-1*), myosin (*myh11*), and transgelin (*tagln*), or of intracellular signaling pathways, such as Ras homolog member G (*rhog*), a novel protein similar to serum/glucocorticoid regulated kinase (*sgk*) also found in zebrafish, and inositol monophosphatase 3 (*impa3*). Proteolytic complexes and enzymes, such as protein arginine methyltransferase (*prmt1*), acetyl-coenzyme A acyltransferase 2 (*acaa2*), and creatine kinase (*ckb*), and the putative homeodomain transcription factor 1 (*phtf1*), transducer of ERBB2 (*tob1a*), neurexin 1a (*nrxn1a*) and thrombospondin 4b (*thbs4b*) were also up-regulated in vitellogenic ovaries.

Two transcripts similar to CDC-like kinase 2 (*si:ch211-81a5.7*) and CD53 cell surface glycoprotein (*zgc:64051*), as well as alanine-glyoxylate aminotransferase (*agxt*), were the most down-regulated genes during vitellogenesis. Other down-regulated genes included the actin-binding protein scinderin (*scin*), mitochondrial enzymes, such as NADH dehydrogenase (ubiquinone) 1 alpha subcomplex subunit 11 (*ndufa11*) and a dihydrodipicolinate synthase-like enzyme, proteolytic complexes and enzymes, as proteasome subunit beta type-9 precursor (*psmb9a*) and carboxypeptidase H (*cph*), a hypothetical protein-encoding gene also found in *Xenopus laevis *(*LOC735233*), and a coiled-coil domain containing 90B novel product (*ccdc90b*).

### Ovarian maturation

Microarray analysis detected 26 up-regulated and 20 down-regulated transcripts in maturing/mature ovaries relative to vitellogenic ovaries, and 32 transcripts could be annotated (Table 2). The most highly up-regulated transcript corresponded to an EST showing sequence similarity to the amphioxus (*Branchiostoma floridae*) *BRAFLDRAFT_128798 *gene, which encodes an hypothetical protein with inferred cysteinyl-tRNA aminoacylation activity. However, the BLAST E-value for the similarity of Senegalese sole clone pgsP0012B12 to this protein was relatively low (2E-14), and therefore conclusive annotation will require the cloning of the sole full-length cDNA. Other highly up-regulated transcripts encoded Na^+^/K^+^-ATPase subunits, such as the beta subunit 1a (*atp1b1a*), alpha subunit 1 (*atp1a1*) and another isoform of the beta subunit (*atpb*), and alpha-2-macroglobulin (*a2m*).

Cytoskeletal proteins, myosin 10 (*myo10*) and type II keratin E3-like protein, and proteins involved in transcriptional and translational responses, such as makorin RING zinc finger protein 1a (*mkrn1*) and ribosomal protein L36 (*rpl36*), were other up-regulated transcripts in mature ovaries. Interestingly, a regulator of vesicular traffic, novel protein similar to vertebrate ADP-ribosylation factor 4 and extended synaptotagmin-2-A (*e-syt2-a*), was also up-regulated. Other transcripts were retinoic acid receptor responder protein 3 (*rarres3*), thioredoxin interacting protein (*txnip*), mitogen-activated protein kinase p38delta (*mapk13*), cytochrome c oxidase subunit I (*cox1*), UDP-glucose dehydrogenase (*ugdh*), and myeloid-associated differentiation marker homolog (*myadm*). Some transcripts that were up-regulated during vitellogenesis showed a further increase during maturation, such as *tob1a *and *nd1*.

During ovarian maturation, more genes appeared to be down-regulated than during vitellogenesis. Among those transcripts, we found type II Na/Pi cotransport system protein (*slc34a2a*) and enzymes such as succinate dehydrogenase complex subunit B (*sdhb*) and fructose-1,6-bisphosphatase (*fbp1*), involved in carbohydrate metabolism, and *impa3*, which was up-regulated during vitellogenesis. Transcript abundance was also reduced for some components and regulators of the cytoskeleton, such as myosin binding protein H (*mybph*), gelsolin (*gsna*), and centaurin delta 2-like (*LOC100005008*). Other transcripts were thrombin (*f2*), monocytic leukemia zinc finger protein (*myst3*), 14-3-3 protein epsilon (*ywhae*), an apolipoprotein L-like protein (*LOC100150119*), zona pellucida C2 (*zpc2*) and histone H2B (*hist1h2be*). These mRNAs are potentially implicated in proteolysis (*f2*), transcription and signal transduction regulation (*myst3 *and *ywhae*, respectively), lipid transport (*LOC100150119*), formation of the zona radiata (*zpc2*), and chromatin compaction (*hist1h2be*).

### Follicular atresia

The comparison of ovaries undergoing follicular atresia *vs*. vitellogenic and mature ovaries revealed the up- and down-regulation of 10 and 16 transcripts, respectively, and 18 transcripts could be annotated (Table 3). One of these transcripts (GenBank accession number FF286365), which was the most highly up-regulated, had sequence similarity to two unknown predicted proteins from gilthead sea bream (*Sparus aurata*) and the puffer fish *Tetraodon nigroviridis *(BLAST E-values of 9E-08 and 3E-04, respectively). This EST apparently encoded a full-length polypeptide which shared 25% identity with a protein named gastrula-specific embryonic protein 1 found in the orange-spotted grouper (*Epinephelus coioides*). The corresponding cDNA clone (pgsP0015C05) was then sequenced in full-length, and the presence of conserved motifs in its deduced amino acid sequence was investigated. These analyses, together with a preliminary phylogenetic reconstruction, clearly indicated that sole FF286365 encoded an ortholog of apolipoprotein C-I (*apoc1*) (Additional file 2). The nucleotide and amino acid sequence of this cDNA was deposited in GenBank with accession number EU835856.

Other transcripts also significantly up-regulated in atretic ovaries were leukocyte cell-derived chemotaxin 2 (*lect*), thrombospondin (*thbs*), heme-binding protein 2 (*hebp2*), apolipoprotein A-I (*apoa1*), S100-like calcium binding protein (*s100*), and enolase (*eno3*). The S100-encoding EST (pgsP0020M08) was a full-length cDNA which allowed further analysis of its deduced amino acid sequence. The analysis indicated that this transcript belongs to the S100a10 subgroup of the EF-Hand calcium-binding proteins superfamily (Additional file 3).

Regarding down-regulated transcripts, *BRAFLDRAFT_128798 *and *a2m *showed the strongest repression in atretic ovaries, which interestingly were highly up-regulated in mature ovaries. Other reduced transcripts were potentially involved in the organization of Golgi complex, such as C-terminal binding protein 1 (*ctbp1*) and Golgi membrane protein 1 (*golm1*), or the telomeric region, such as a novel protein similar to vertebrate RAP1 interacting factor homolog (*rif1*), as well as in transcription and translation regulation, such as elongation factor 1 alpha (*ef1a*) and zinc finger protein 576 (*znf576*). The identity of sole FF288651 and FF282343 as *ef1a *and *znf576*, respectively, was however not conclusive since the BLAST E-values were low. Cytoplasmic FMR1 interacting protein 1 homolog (*cyfip1*) and elongation of very long chain fatty acids protein 1 (*elovl1b*), which may be involved in the control of cell projections and fatty acid biosynthesis, respectively, were also down-regulated.

### Validation of microarray data by real-time qPCR

**Figure 4:**
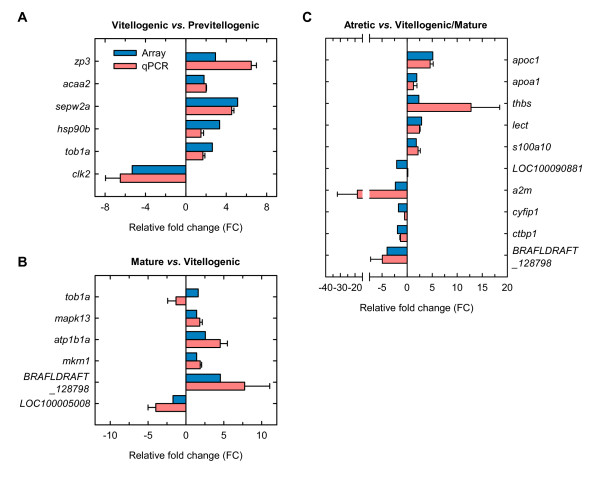
**Real-time qPCR validation of differential expression in vitellogenic *vs*. previtellogenic ovaries (A), mature *vs*. vitellogenic ovaries (B), and atretic *vs*. vitellogenic/mature ovaries (C)**. Bar graphs represent relative fold change (FC) of selected transcripts obtained with the microarray (black bars) or by qPCR (with bars). The mean FC ± SEM are shown for each gene. The qPCR and microarray experiments were both conducted on RNA extracted from the same three female individuals for each developmental stage examined. See Tables 1, 2 and 3 for transcript abbreviations.

A number of differentially expressed ESTs (*n *= 20) in vitellogenic, mature and atretic ovaries were further selected to verify the changes in expression by real-time quantitative RT-PCR (qPCR). The expression of all twenty genes followed the same pattern whether evaluated by microarray or qPCR (Figure 4). Two genes, *tob1a *and *LOC100090881*, were however an exception. For *tob1a*, a significant increase during maturation observed with the microarray could not be detected (*p *= 0.78) by qPCR (Figure 4B and 4C), whereas the significant down-regulation of *LOC100090881 *during atresia could not be confirmed (*p *= 0.88) by qPCR (Figure 4C). All other genes showed in general a similar relative expression pattern by both microarray and qPCR, resulting in an overall success rate of 91% (2 inconsistencies out of 22 comparisons, since *tob1a *and *BRAFLDRAFT_128798 *were significantly regulated both during vitellogenesis and atresia by microarray analysis). For *a2m*, however, the FC determined with the array (2.35) was about 10 times lower than that measured by qPCR (18.38), which is a known phenomenon observed in oligo-arrays when background subtraction is not performed (as in the present study) [32]. Usually, a two-fold change is considered as the cut-off around which microarray and qRT-PCR data begin to loose correlation [33]. Finally, few transcripts that did not show significant differences in expression levels with the microarray were also selected for qPCR. These analyses did not show significant changes in the expression level consistent with the array data (data not shown).

### Differential expression during follicular atresia

**Figure 5:**
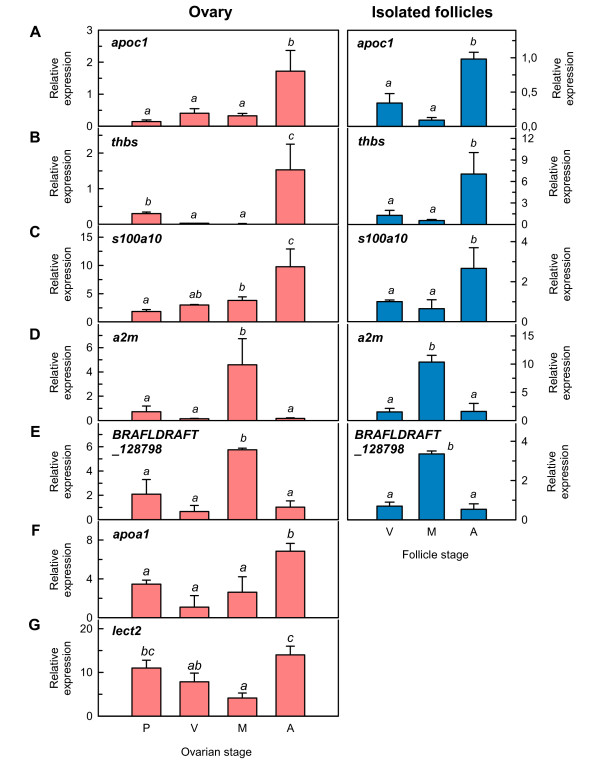
**Expression profile of selected transcripts during Senegalese sole ovarian development**. Histograms represent relative mean expression values ± SEM (*n *= 3 females) in ovaries (left panels) or in isolated ovarian follicles (right panels) of apolipoprotein C-I (*apoc1*; A), thrombospondin (*thbs*; B), S100A10 calcium binding protein (*s100a10*; C), alpha-2-macroglobulin (*a2m*; D), hypothetical protein BRAFLDRAFT_128798 (*BRAFLDRAFT_128798*; E), apolipoprotein A-I (*apoa1*; F), and leukocyte cell-derived chemotaxin 2 (*lect2*; G). Data were determined by qPCR and normalized to 18S ribosomal protein (*18S*) expression. Data with different superscript are statistically significant (*p *< 0.05).

Some regulated transcripts in atretic ovaries relative to mature/vitellogenic ovaries, such as *apoc1*, *apoa1*, *thbs*, *lect2*, *s100a10*, *a2m *and *BRAFLDRAFT_128798*, were further analyzed by qPCR to investigate how broadly they might be expressed during ovarian development (Figure 5). For *apoc1*, *thbs*, *s100a10*, *a2m *and *BRAFLDRAFT_128798*, these analyses were also carried out on manually isolated ovarian follicles at the stages of vitellogenesis, maturation and atresia. The results confirmed that *apoc1*, *apoa1*, *thbs*, *lect2 *and *s1001a10 *transcripts were significantly (*p *< 0.05) up-regulated in atretic ovaries, whereas *a2m *and *BRAFLDRAFT_128798 *transcripts were accumulated in mature ovaries and strongly down-regulated in atretic ovaries, thus demonstrating the same expression pattern as that observed with the microarray. The data also revealed that *apoa1*, *thbs *and *lect2 *showed relatively high relative expression levels in previtellogenic ovaries in addition to during atresia.

### Cellular localization of differentially expressed genes

**Figure 6:**
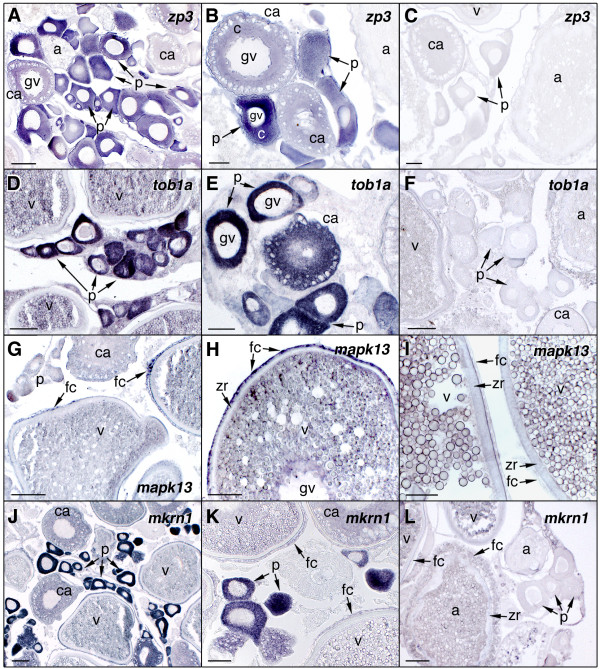
**In situ hybridization of zona protein 3 (*zp3*), transducer of ERBB2 (*tob1a*), mitogen-activated protein kinase p38delta (*mpk13*), and makorin RING zinc finger protein 1a (*mkrn1*) transcripts in the Senegalese sole ovary**. Ovarian histological sections were hybridized with antisense digoxigenin-labeled riboprobes for *zp3 *(A and B), *tob1a *(D and E), *mpk13 *(G and H) and *mkrn1 *(J and K). The hybridization signal is colored dark-blue to purple. No staining signal was observed using sense probes (C, F, I and L). gv, germinal vesicle; p, previtellogenic ovarian follicle; ca, ovarian follicles with oocytes at the cortical-alveolus stage; c, oocyte cytoplasm; a, atretic ovarian follicle; zr, zona radiata; fc, follicular cells. Bars, 50 μm (B, C, D, E, F, H, I, and K), 100 μm (A, G, J and L).

**Figure 7:**
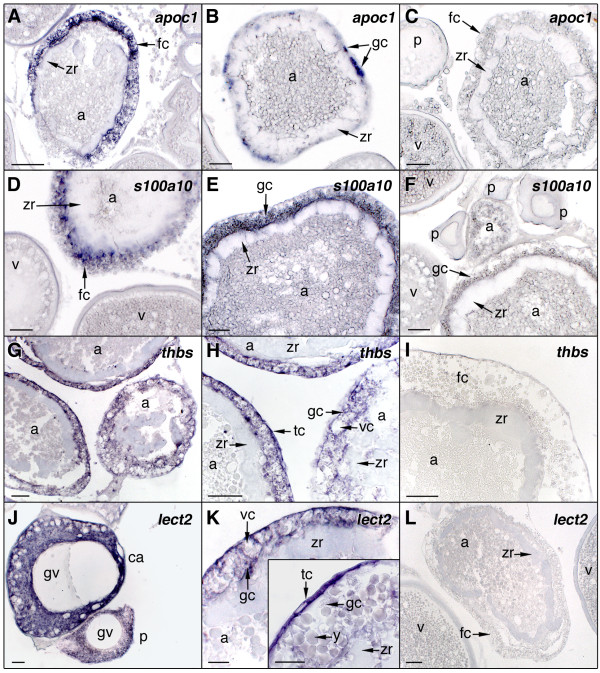
**In situ hybridization of apolipoprotein C-I (*apoc1*), S100A10 calcium binding protein (*s100a10*), thrombospondin (*thbs*), and leukocyte cell-derived chemotaxin 2 (*lect2*) transcripts in the Senegalese sole ovary**. Ovarian histological sections were hybridized with antisense digoxigenin-labeled riboprobes for *apoc1 *(A and B), *s100a10 *(D and E), *thbs *(G and H), and *lect2 *(J and K). No staining signal was observed using sense probes (C, F, I and L). gc, granulosa cells; tc, theca cells; vc, vacuole in granulosa cells; y, yolk granules. Other abbreviations as in Fig. 5. Bars, 20 μm (J, K), 50 μm (B, C, D, E, F, G, H, I and L), 100 μm (A).

To determine the cell type-specific expression of representative transcripts in the ovary, in situ hybridization was carried out on ovarian histological sections using specific antisense riboprobes. For these experiments, we selected transcripts that were up-regulated in vitellogenic and mature ovaries, *zp3*, *tob1a*, *mapk13 *and *mkrn1 *(Figure 6), or in atretic ovaries, *apoc1*, *s100a10*, *thbs *and *lect2 *(Figure 7). The *zp3 *hybridization signal was weakly detected in the cytoplasm of previtellogenic oocytes, whereas the signal increased in early cortical alveolus stage oocytes to subsequently diminished again at later stages (Figure 6A and 6B). The staining was absent in vitellogenic oocytes as well as in the surrounding follicle cell layers. A similar localization pattern was observed for *tob1a *(Figure 6D and 6E) and *mkrn1 *(Figure 6J and 6K), although their hybridization signals remained visible, but much weaker, in the cytoplasm during vitellogenesis. A weak *mkrn1 *staining was also seen in follicular cells of vitellogenic follicles. *mapk13 *transcripts were exclusively localized in the surrounding follicular cells of late vitellogenic oocytes, whereas expression in ovarian follicles at other stages was not consistently detected (Figure 6G and 6H). For all these transcripts, sense probes resulted in no signal (Figure 6C, F, I and 6L).

Regarding the transcripts up-regulated during ovarian atresia, *apoc1*-specific antisense probes showed an intense and specific staining in hypertrophied and vacuolized follicular cells of atretic follicles, which was increasing as follicular atresia progressed (Figure 7A and 7B). The same staining pattern was found for *s100a10 *(Figure 7D and 7E) and *thbs *(Figure 7G and 7H). The *lect2 *transcripts were found in theca cells of atretic follicles (Figure 7K inset) but a weaker and more diffuse staining was also detected in hypertrophied granulosa cells (Figure 7K). Primary growth oocytes, including cortical alveolus stage oocytes, also expressed *thbs *and *lect2 *mRNAs (Figure 7J), in agreement with their increased levels previously found in previtellogenic ovaries by qPCR (Figure 5B and 5G). Sense probes for all of these transcripts were negative (Figure 7C, F, I and 7L).

## Discussion

The present work has identified a number of differentially expressed genes in the Senegalese sole ovary that may play different roles during ovarian follicle growth and maturation, as well as several genes that were not previously found to be regulated in the teleost ovary. The expression of some genes specifically in follicular cells of atretic follicles suggest the role of these cells in the activation of molecular pathways associated with ovarian follicle atresia which have not been previously recognized in fish.

### Microarray performance

In this study, a first-generation Senegalese sole oligonucleotide microarray was employed. This array represents the second high-density microarray available for commercial flatfish, in addition to that recently published for Atlantic halibut (*Hippoglossus hippoglossus*) [34], and has been previously shown to perform well to detect differences in gene expression [27].

Transcriptome analysis during ovarian growth, maturation, and follicular atresia in Senegalese sole showed the differential expression of 118 genes. This number of regulated genes is lower than that reported in similar studies on trout ovaries by using cDNA microarrays [13,15] or SSH [17], where changes in the expression of up to 600 genes have been reported. However, our data are more similar to the expression profiling obtained from the comparison of halibut larval stages not very distant during development (e.g., mouth opening *vs*. post-hatch) by using an oligo microarray (44 differentially expressed genes in Atlantic halibut [34]). The apparent discrepancy in the overall number of genes regulated during ovarian development observed in this work with respect to published reports in salmonids using cDNA microarrays may be related to the limited number of unique genes represented in our array when compared with the salmonid platforms, or to the fact that oligo arrays are usually more stringent than cDNA arrays [35]. Another important aspect that can be considered is that salmonids have synchronous ovaries, unlike the Senegalese sole that has a group-synchronous ovary, and therefore ovarian follicles at different developmental stages are present at any time during the spawning season.

### Folliculogenesis and oocyte growth

The period of ovarian vitellogenesis in fish is mainly regulated by the follicle-stimulating hormone (FSH) and involves the differentiation and growth of ovarian follicles mainly by the incorporation of circulating vitellogenins and very low-density lipoproteins (VLDL) in the oocyte [36]. Among the genes regulated in Senegalese sole vitellogenic ovaries, the GO terms overrepresented belong to the metabolic, cellular, biological and developmental processes categories, and this is consistent with the rapid rates of growth and development of the ovarian follicles at this stage. Thus, transcripts possibly related to mitochondrial energy production (*cox1*, *cytb*, *nd3*, *nd1, acca2*), cystoskeleton formation and organization (*bactin1*, *actc1l*, *tagln*, *tpm1-1*, *krt8*, *myh11*), intracellular signaling pathways (*rhog*, *sgk*), and cell-to-cell and cell-to-matrix interactions (*mibp2*, *thbs-4b*, *zgc:64051*), that may play different roles during the formation and growth of the ovarian follicles, were over-expressed relative to previtellogenic ovaries. Also, as seen in previous ovarian transcriptome studies in salmonids and tilapia [15,17,37], as well as in zebrafish fully-grown ovarian follicles [12], zona radiata (*zp3*) and *hsp90b *transcripts were strongly up-regulated. Vertebrate members of the heat shock protein 90 family play a post-translational regulatory role within the cell by interacting with several important cellular signalling molecules and transcription factors, such as steroid receptors, modulating their activity [38]. High abundance of *hsp90b *transcripts is a common feature of mammalian, fish and *Drosophila *ovaries and eggs [12].

During oocyte growth, meiosis is arrested at prophase I, and it will not proceed until it is activated by the maturation promoting (MPF). The MPF is a cytoplasmic complex specifically formed during oocyte maturation consisting of cyclin B and Cd2, a serine-threonine protein kinase [39]. Studies in zebrafish have shown that immature oocytes contain Cd2 proteins but not cyclin B, and therefore the absence of cyclin B translation is likely the main mechanism to maintain meiosis arrest [40]. However, the roles of other cyclins (e.g., cyclin A) and protein kinases during this process in growing fish oocytes is poorly known. It is therefore of interest the strong repression of a transcript similar to vertebrate CDC-like kinase 2 (*si:ch211-81a5.7*) in sole vitellogenic ovaries.

The vitellogenic period is also characterized by intense deposition of RNA and proteins, as well as lipids, vitamins and hormones, which are necessary during the earliest steps of embryonic development [41]. Maternal RNAs are produced endogenously by the oocyte and stored during oogenesis, and they become usable for embryogenesis upon egg activation and fertilization, usually sometimes after a process of activation involving translation or protein modification [42]. One of these transcripts found in the Senegalese sole ovary, and highly expressed in previtellogenic oocytes of vitellogenic ovaries, was *tob1a*. Interestingly, the array and qPCR analyses detected the up-regulation of *tob1a *only in vitellogenic ovaries, which may suggest that this transcript starts to be accumulated in previtellogenic oocytes at the onset of vitellogenesis. *tob1a *is also found in trout ovaries [15] and encodes a transcriptional repressor of the BTG/Tob family of antiproliferative proteins [43]. Tob1a plays an important role during embryonic dorsoventral patterning in zebrafish by inhibiting transcriptional regulation stimulation by β-catenin, a factor that is essential for the dorsal development of amphibian and fish embryos [44]. Other transcripts that may also be stored in oocytes are *nrxn1a *and *mkrn1*. *nrxn1a *is a member of a family of adhesion molecules involved in the formation and function of synapses. In both zebrafish and amphibians [45,46], neurexins genes are expressed in the ovary and in embryos before the activation of zygotic transcription, and interestingly, a parental origin of some of the embryonic neurexin isoforms has been suggested [45]. The *mkrn1*, as *tob1a*, was detected at high levels in Senegalese sole previtellogenic oocytes of vitellogenic ovaries. This transcript encodes a putative ribonucleoprotein with a distinctive array of zinc finger domains that may play an important role in embryonic development and neurogenesis, as reported for the amphibian makorin-2 [47].

The most highly up-regulated transcript in sole vitellogenic ovaries showed sequence similarity to tilapia selenoprotein W2a (*sepw2a*). Selenoproteins are a diverse group of proteins, with 25 members in humans [48], that contain selenocysteine (Sec) which is incorporated by a redefined in-frame UGA codon and requires the involvement of a complex translational machinery [49]. Selenoproteins with characterized functions are enzymes involved in redox reactions, such as glutathione peroxidase, thioredoxin reductase, and iodothyronine deiodinase, and thus they are believed to protect the cells from oxidative damage and apoptosis [49]. Expression of selenoproteins in fish ovaries has also been reported in salmonids [15] and tilapia [37]. These coincident findings suggest that seleneproteins might be accumulated in fish follicles for protection against oxidative stress during folliculogenesis and oocyte growth. In mammals, selenium stimulates proliferation of granulosa cells from small follicles and also potentiates FSH induction of estradiol secretion [50]. In addition, accumulation of maternal selenoproteins in ovarian follicles may have a role for antioxidant protection of the offspring [50].

### Oocyte maturation and hydration

During the maturation of follicle-enclosed oocytes, meiosis is reinitiated in response to progestagens produced by the follicular cells after luteinizing hormone (LH) stimulation [39]. During this process, the germinal vesicle migrates towards the oocyte periphery, the nuclear envelope breaks down, the first meiotic division occurs, and the chromosomes proceed to second meiotic metaphase where they arrest; at this point, the oocyte will ovulate and becomes an egg [51]. Oocyte maturation is also accompanied by important changes in the cytoarchitecture and function of the ovarian follicle, since steroidogenic pathways in granulosa cells are switched from estrogen to progestagen production, intercellular communication oocyte-granulosa cells is resumed, and the zona radiata becomes more compacted [39,52,53]. At this stage, ovarian transcripts associated with the regulation of intracellular signalling pathways (such as *mapk13 *in follicular cells, and *ywhae*), and cystoskeleton (*mybph*, *gsna*, *myo10*, *LOC100005008*), as well as in the assembly of the nucleosome (*hist1h2be*, *myst3*), were significantly regulated in Senegalese sole, which is consistent with the important nuclear and cytoplasmic changes occurring in the ovarian follicle during oocyte maturation.

One transcript overexpressed in mature ovaries was *e-syt2-a *which is related to the synaptotagmin family of vesicle proteins that are believed to function as calcium sensors for vesicle exocytosis at synapsis [54]. It is known that fish oocytes, as the eggs of most organisms, suffer a transient elevation of intracellular free Ca^2+ ^following fertilization, an event that triggers a series of biochemical pathways required for the block of polyspermy, activation of metabolism, re-entry into the cell cycle, and execution of the developmental program [55]. One of the earliest responses to the C^2+ ^wave in the oocyte is the cortical alveoli exocytosis wich produces an elevation of the chorion and the separation of the egg surface [55]. Studies in mammalian eggs suggest that the release of cortical granules in mature eggs is dependent upon calcium-dependent synaptosome-associated protein 25 (SNAP-25) which might be regulated by binding to Ca^2+^-dependent synaptotagmins as it occurs in neurons [56,57]. These observations therefore suggest that the induction of *e-syst2-a *transcripts in sole mature ovaries might be part of the molecular pathways activated in the oocyte in preparation for fertilization.

In marine teleost that produce buoyant (pelagic) eggs, such as the Senegalese sole, oocytes continue to enlarge during maturation owing to hydration [58]. The hydrolysis of the oocyte yolk proteins by the lysosomal proteases cathepsin B (Catb) and/or cathepsin L (Catl) that occurs during maturation results in the increase of free amino acids in the ooplasm, incrementing the osmotic pressure of the oocyte, and hence facilitating water uptake mediated by aquaporin-1b (Aqp1b) [58-60]. Current evidence in some fish species suggest that both Catb and Aqp1b are regulated post-translationally rather than transcriptionally in oocytes during meiotic maturation [61-63]. Accordingly, in the present study, we did not detect changes in the expression levels of *aqp1b *and *catl*, as well as of other cathepsins and proteases, for which specific oligos were present in the microarray. However, probes for *catb *were lacking, and therefore the regulation of this transcript during oocyte maturation in Senegalese sole can not be ruled out. Nevertheless, the up-regulation of *zgc:77650*, a transcript showing sequence similarity to a GTP-binding protein (ADP-ribosylation factor 4) involved in protein trafficking that may modulate vesicle budding and uncoating within the Golgi apparatus [64], it is of interest. This observation may indicate a role of vesicle trafficking during oocyte maturation that might be important, for instance, in the delivery of lysosomal cathepsin to yolk granules or in the control of Aqp1b shuttling into the oocyte plasma membrane. These mechanisms are however not yet elucidated in fish oocytes and need to be investigated in the future.

The accumulation of inorganic ions in oocytes undergoing maturation, mainly K^+ ^and Na^+^, may account for about 50% of the final osmotic pressure, and therefore it is considered as an additional mechanism mediating fish oocyte hydration [59]. Interestingly, we found that three of the most highly up-regulated transcripts in mature ovaries corresponded to Na^+^-K^+^-ATPase subunits (*atp1b1a*, *atp1a1 *and *atpb*), whereas the solute carrier *slc34a2a*, also known as the type II Na^+^/Pi cotransporter, was one of the most down-regulated transcripts. In trout, *slc26 *(Na^+^-independent chloride/iodide transporter) and aquaporin-4 (*aqp4*) were found to be overexpressed in ovarian tissue at maturation [13]. The causes for the different ion and water transport-associated transcripts regulated during oocyte maturation in sole and trout are intriguing, although the fact that trout oocytes exhibit a much lower hydration than Senegalese sole oocytes, resulting in the production of demersal eggs, may be one of the reasons.

In vertebrates, retinoic acid regulates the transcription of many genes involved in embryonic development and germ cell differentiation through binding to nuclear receptors (retinoic acid receptors, RARs and retinoid × receptors, RXRs) [65]. In mammals, retinoic acid also affects the acquisition of developmental competence of oocytes and the steroidogenesis of ovarian follicle cells [66,67]. In fish, recent studies in trout suggest that follicular cells express several genes associated with retinoid and carotenoid metabolism indicating the presence of an additional pathway to provide retinoids to the oocyte in addition to the receptor mediated uptake of lipoproteins [68]. A transcript related to this system was induced in Senegalese sole mature ovaries, *rarres3*, which shows sequence similarity to the tazarotene (synthetic, topical retinoid)-induced gene 3 (TIG3; Retinoic Acid Receptor Responder 3). This gene encodes a growth regulator that possibly mediates some of the growth suppressive effects of retinoids [69]. Although the cell localization of *rarres3 *in the ovarian follicle was not determined here its overexpression in the mature sole ovary may indicate that retinoids could play an additional paracrine role by affecting the expression of suppressor/growth regulatory pathways in the ovary.

Another gene that could play a paracrine role in the sole ovary at the maturation stage is the proteinase inhibitor *a2m*. In the mammalian ovary, *a2m *modulates the actions of growth factors and cytokines, and recent works suggest that it may have autocrine or paracrine roles in granulosa cells potentially important for regulation of estradiol production and development of dominant follicles [70]. Interestingly, we observed that the expression of this mRNA was specifically up-regulated during maturation, whereas in atretic ovaries its induction was prevented. In contrast, down-regulation of *a2m *was reported in trout precocious mature ovaries [15].

Finally, similarly to that described in trout ovaries at the time of meiosis resumption, we found high levels of coagulation factor II (thrombin I), *f2*, in mature ovaries. In trout, overexpression of the coagulation factor V (*cf5*) has been speculated to be related with the prevention of bleeding from ruptured ovarian follicles at the time of ovulation [13]. Although in Senegalese sole we found the induction of an apparently different coagulation factor, a similar scenario may be considered to occur in the flatfish ovary.

### Follicular atresia

Ovarian atresia is a common phenomenon in teleosts under both natural and experimental conditions during which a number of vitellogenic ovarian follicles fail to complete maturation and ovulation, degenerate and are eventually reabsorbed [19,20]. Ovarian follicle atresia in fish seems not to be mediated by apoptosis in the follicular cells, unlike in mammals, and thus this process appears to be different than the post-ovulatory follicular reabsorption mechanism, which is apparently mediated by apoptosis [22,71,72]. Therefore, apoptosis may not be relevant at the onset of atresia, although it may contribute to a more efficient removal of atretic follicles during ovarian follicular regression after spawning [73,74]. The ovarian transcriptome analysis in Senegalese sole might support this view since the expression levels of none of the apoptosis-related genes that were represented in the microarray, identified by GO annotation, changed in atretic ovaries related to vitellogenic/mature ovaries.

In Senegalese sole, as in other teleosts [19,20], the process of ovarian follicle atresia and resorption is preceded by marked morphological changes in both the oocyte and follicular cells, such as the disintegration of the oocyte germinal vesicle and of other cytoplasmic organelles, the fragmentation of the zona radiata, and the hypertrophy of the granulosa cells. These cells become phagocytic with digestive vacuoles and incorporate and digest the oocyte yolk as well as other oocyte components and organelles, and they may also secrete enzymes which digest the yolk [19,20,73,75]. In atretic ovaries, two of the up-regulated genes corresponded to *apoa1 *and *apoc1*, which are part of chylomicrons, very low density lipoproteins (VLDL) and high density lipoproteins (HDL) involved in lipid transport in the bloodstream [76]. In addition, we found reduced *elovl1b *transcripts possibly involved in the control of the synthesis of very long chain fatty acids and sphingolipids in the ovary [77]. Studies in rainbow trout have shown that during the course of follicular atresia there is a massive transfer of the oocyte yolk proteins, and possibly lipids, into the bloodstream combined with HDL [78], as a result of the ingestion and digestion of the yolk by the follicular cells [19,20]. The finding of high *apoa1 *and *apoc1 *transcript levels in hypertrophied follicular cells (at least for *apoc1*) of Senegalese sole, together with that recently reported for the fatty acid-binding protein 11 (*fap11*) [79], provides additional evidence for this mechanism in teleosts. However, the nature of the numerous invasive *apoc1*- and *fabp11*-expressing cells (theca or granulosa cells) remains to be clearly established. Nevertheless, these data suggest the importance of lipid-metabolic processes during follicular atresia in fish [79], which may have evolved to facilitate the redistribution of energy-rich yolk materials from oocytes that fail to develop properly [80].

In humans, *Apoc-I *is primarily expressed in the liver but also in the lung, skin, spleen, adipose tissue, and brain [81]. ApoC-I can interact with lipid surfaces and play an important role in controlling plasma lipoprotein metabolism by the regulation of several enzymes, such as lipoprotein lipase or phospholipase A2 [76]. The expression of *ApoC-I *in the mammalian ovary has not been reported, unlike that of *ApoA-I *and *ApoE *which are expressed by luteinizing granulosa cells and theca cells, respectively, of atretic follicles [82,83]. Intraovarian ApoE controls theca cell production of androgens as well as limiting the size of the theca cell compartment [83]. Teleost ovary and embryos also express an ApoC-I ortholog as it has been recently shown [18,84,85] and confirmed in the present study, although its function is largely unknown. In the embryo, *apoc1 *is localized in the yolk syncytial layer [85], along with *apoe*, *apoa1 *and *apo14 *[86,87], suggesting its role in the nutrition of the developing embryo through the synthesis and secretion of apolipoproteins and lipoproteins. Therefore, the expression of *apoc1 *in follicular cells of fish atretic follicles, which has not been previously reported, may have a similar role for the resorbption of lipids and lipoproteins stored in the oocyte. Interestingly, trout eggs obtained by hormonal induction, which result in alevins with a high percentage of morphological abnormalities at the yolk-sac resorption stage, also show a dramatic increase of *apoc1 *[18]. Altogether, these findings suggest that ApoC-I could be a useful marker to identify factors involved in premature ovarian regression and abnormal embryo development in cultured fish. It is worth noting that in humans it has been recently proposed that serum ApoC-I may be useful for early demonstration of metabolic abnormality in women with polycystic ovary syndrome [88].

In histological sections of Senegalese sole ovarian follicles at advanced atresia, we observed the presence of blood cells such as erythrocytes and leukocytes, possibly derived from the ovarian stroma and/or the theca, which invaded the degenerating oocyte. The presence of granulocytes (polymorphonuclear leukocytes) in atretic follicles is reported in other fish species [21,89,90], and suggest a relationship between follicular regression and immune cells [73]. The specific function of immune cells (eosinophilic granulocytes and macrophages) during follicular atresia in fish is not well known, although it has been proposed that they may act synergistically with follicular cells in the resorption of the oocyte by releasing their granules containing lytic enzymes [89]. In the mammalian ovary, the leukocyte-ovarian cell interactions through the release of chemokines is believed to play an important role for leukocyte recruitment and activation during follicular atresia, ovulation and luteal function [91]. In teleosts, however, the molecular mechanisms mediating the invasion of immune cells in atretic follicles are largely unknown. In the present study, we noted high *lect2 *expression levels in theca cells of atretic follicles, a transcript related to mammalian *Lect2 *which encodes a protein with chemotactic properties for human neutrophils [92]. This observation may provide evidence for the presence of a chemotaxin-mediated mechanism for leukocyte accumulation in fish follicles at advanced atresia, similarly to that occurs during the formation of the corpus luteum in the mammalian ovary [91]. However, whereas a number of different chemokines have been found in the mammalian ovary [91], ovarian expression of *Lect2 *has not been yet reported, and therefore the structural and functional relationships of Senegalese sole *lect2 *with other ovarian chemokines requires further investigation.

In addition to *lect2*, we also found high expression levels of the calcium binding protein-encoding gene *s100a10 *in atretic follicles. In mammals, S100 proteins are localized in the cytoplasm and/or nucleus of a wide range of cells and are involved in the regulation of a number of cellular processes such as cell cycle progression and differentiation [93]. In the ovary, S100a10 plays an antiapoptotic function by binding the Bcl-xL/Bcl-2-associated death promoter and its expression in granulosa cells is stimulated by gonadotropins and follicle survival factors, including the epidermal growth factor, the basic fibroblast growth factor, and interleukin-1β [94]. It is known, however, that some S100 proteins can also be released into the extracellular space and act as chemoattractants for leukocytes or activators of macrophages [93]. Therefore, the high expression levels of *s100a10 *in sole atretic follicles may play a dual function, to protect follicular cells from apoptosis during atresia and to act as chemoattractant for leukocytes and macrophages. In support of this hypothesis is the reported down-regulation of an S100 homologue in trout post-ovulatory follicles [95], which based on our phylogenetic analysis is in fact an *s100a10 *ortholog.

The mammalian ovary is distinctive in that it is a tissue that undergoes physiological angiogenesis, in which blood vessels are programmed to develop and regress in a cyclic manner [96]. This mechanism is tightly regulated by pro- and antiangiogenic factors such as the members of the thrombospondin (TSP) family TSP-1 and TSP-2, which are among the naturally occurring inhibitors of angiogenesis. These secreted proteins are expressed by granulosa cells of atretic follicles and in the corpus luteum after ovulation in rats and primates [96,97], suggesting that they may be involved in the cessation of angiogenesis in follicles undergoing atresia [96]. Our microarray analysis revealed that a thrombospondin isoform (*thbs*), distinct from *thbs4b *which was overexpressed in vitellogenic ovaries, was up-regulated in Senegalese sole follicular cells during follicular atresia. *thbs *was similar to mammalian TSP-1 and TSP-2, which indicates that the inhibition of angiogenesis may be an important mechanism regulating atresia in both mammalian and fish ovarian follicles. However, primary growth oocytes also expressed *thbs *which may point to an additional role of this protein during folliculogenesis and/or as a maternal molecule for early embryonic development.

In contrast to apoptosis, Wood and Van Der Kraak [22,80] proposed that proteolytic degradation of yolk proteins, mediated by the differential activation of Catl in the oocyte, may be the initial event leading to follicular atresia in fish. In the present study we did not detect changes in the expression of *catl *transcripts in atretic ovaries, which may suggest a potential post-transcriptional regulation of this protease during ovarian follicle atresia as discussed earlier. In any event, the specific oocyte mechanisms involved in the regulation of protease activity during atresia in fish, as well as the origin of the signals that presumably activate this system, are still largely unknown and warrant further investigation.

## Conclusion

The present study has contributed to identify differences in gene expression during ovarian development in Senegalese sole despite that, at this point, the microarray platform employed was not ovary-specific and contained a limited number of represented genes. Some of the genes identified were not described before in the teleost ovary, and therefore the present data provide a basis for future studies on their regulation and function. Particularly, determination of the cell-type specific localization of some transcripts suggest the involvement of follicular cells in yolk resorbption, chemotaxin-mediated leukocyte migration, angiogenesis and prevention of apoptosis during ovarian atresia. These observations also indicate that some of the transcripts, such as *e-syt2-a*, *apoc1 *and *s100a10*, may be useful markers contributing to the identification of factors involved in the acquisition of egg fertilization competence and follicular regression. Clearly, however, further experimental studies will be necessary to determine when these mRNAs are translated as well as the physiological functions of the encoded proteins. The future increase in the sequencing of the Senegalese sole ovarian transcriptome will enhance our knowledge on the molecular pathways involved in oogenesis, and will facilitate the comparative genomic analysis of gonad development in teleosts.

## Methods

### Animals and biological samples

Adult Senegalese sole (1219 ± 90g) F1 generation were maintained as previously described [24]. Females (*n *= 3-5) were sacrificed at different times during the annual reproductive cycle, corresponding to different folliculogenesis stages [24,28]. Thus, females with previtellogenic and vitellogenic ovaries were collected during July and November, respectively. Maturing and mature ovaries were collected from females treated with intramuscular injection of 5 μg/kg GnRHa and killed 24-48 h later as described [24]. Finally, atretic ovaries were obtained in April-May, as well as from females treated with GnRHa. At all sampling times, fish were sedated with 500 ppm phenoxyethanol, killed by decapitation, and the body and gonads of each animal weighed to calculate the GSI (gonad weight/body weight × 100). Pieces of the ovaries were immediately removed and placed in Petri dishes containing 10 ml of 75% Leibovitz L-15 medium with L-glutamine (Sigma) and 100 mg gentamicin/ml, pH 7.5 [60]. Follicle-enclosed oocytes at vitellogenesis, maturation and atresia were manually isolated from the rest of the ovary using watchmaker forceps. One piece of the ovary, as well as the isolated ovarian follicles, were deep-frozen in liquid nitrogen and stored at -80°C until RNA extraction. Two additional pieces of the gonad, adjacent to the piece sampled for RNA extraction, were fixed in modified Bouin solution (75% picric acid and 25% formalin) for histological analysis, or in 4% paraformaldehyde (PFA) for in situ hybridization. Procedures relating to the care and use of animals were approved by the Ethics Committee from Institut de Recerca i Tecnologia Agroalimentàries (IRTA, Spain) in accordance with the Guiding Principles for the Care and Use of Laboratory Animals.

### Histological analysis

Ovaries fixed in Bouin solution for 3-4 h were dehydrated, embedded in paraplast, sectioned at 5 μm, and stained with hematoxilin-eosin. Alternatively, fixed gonads were embedded in glycol methacrylate resin (Technovit 7100, Heraeus Kultzer), sectioned at 3 μm and stained with methylene blue/azure II/basic fuchsin. The percentages of previtellogenic, early vitellogenic, vitellogenic, mature and atretic oocytes were calculated by counting 100-150 total ovarian follicles in at least three different histological sections from the same ovary, as previously described [24].

### RNA extraction, microarray hybridization and analysis

Total RNA was extracted from ovaries at previtellogenesis, vitellogenesis, maturation and atresia, determined by histological examination, using the RNeasy extraction kit (Qiagen) and treated with DNAse following the manufacturer's instructions. For each ovarian developmental stage, RNA was extracted from the ovary of three different females. The quality and concentration of the RNA was analyzed using the Agilent 2100 bioanalyzer and NanoDrop™ ND-1000 (Thermo Scientific). Samples with RNA integrity number (RIN; [98]) < 6.0 were discarded. Total RNA (0.5 μg) from each of the twelve samples was amplified and labelled with fluorescent cyanine dyes, Cy3 or Cy5, using the Eberwein mRNA amplification procedure [99] employing the MessageAmp™ aRNA amplification kit from Ambion (Applied Biosystems) following the manufacturer's instructions with minor modifications. The Cy3- and Cy5-labelled aRNAs synthesized from RNAs originated from two different ovarian stages were mixed in equal amounts and hybridized to an oligonucleotide microarray representing 5,087 Senegalese sole unigenes [27]. These 60-mer probes were designed against the 3'end sequences of Senegalese sole expressed sequence tags (ESTs) [27]. Each hybridization was done in triplicate with aRNA from three different females, so three different biological replicates per ovarian stage were analyzed. To estimate the rate of false-positive expression, a self-to-self hybridization was carried out, in which total RNA from two different aliquots of previtellogenic ovaries were used to produce either Cy3 or Cy5 labelled aRNA. Hybridizations were carried out for 17 h at 60°C using Agilent's gaskets G2534-60002, G2534A hybridization chambers, and DNA Hybridization Oven G2545A, according to the manufacturer's instructions.

Microarray raw data were obtained using Agilent's DNA Microarray Scanner G2505B and Feature Extraction software (v10.1). The raw fluorescence intensity data were processed using the Polyphemus™ software (Oryzon Genomics), which includes spatial data compensation, non-significant expressed data filtering, and data normalization. Data normalization was carried out by an improved version of the nonlinear *Q-splines *normalization method [100], enhanced with robust regression techniques. Normalized and log-transformed data were used to calculate the FC values. Differential expression was assessed with Polyphemus™ analyzing biological replicates based on repeated experiments using robust statistics on average technical replicates removing the outlier points (caused by dust or array imperfection). The *p-values *were calculated based on the absolute value of the *regularized t-statistic *[101], which uses a Bayesian framework to derive the algorithm, using internal replicated controls to assess the minimum technical variability of the process. A *p-value *< 0.01 was considered significant. Cut-offs for significant changes were always greater than the inherent experimental variation as assessed by the FC of internal controls and/or self-to-self hybridizations. The microarray data have been deposited in NCBI's [102] Gene Expression Omnibus (GEO) [103] and are accessible through GEO Series accession number GSE17337 [104].

### EST Annotation, gene ontology and amino acid sequence analysis

Annotation of ESTs and extraction of GO terms to each obtained hit using existing annotations was done using the BLAST2GO v1 program [31] as previously described [27]. ESTs that were considered significantly expressed after microarray analysis, but that they could not be initially annotated, were sequenced from the 5' and 3' ends using an ABI PRISM 377 DNA analyzer (Applied Biosystems). Basic local alignment search tool (BLAST) software [102], employing the 5'end nucleotide sequences or the consensus 5' and 3' end sequence, was used to re-annotate these ESTs. For some of these ESTs, further annotation required the search for conserved motifs using PROSITE [105] and multiple amino acid sequence alignments using ClustalW [106]. The neighbor-joining (NJ) phylogenetic analysis [107] of the amino acid alignments was also carried out based on mean character distances. The robustness of the phylogenetic tree was tested by bootstrap analysis [108] with 1000 repetitions.

### Real-time quantitative RT-PCR

The relative mRNA levels of selected ESTs during ovarian development were determined by qPCR on the same RNA samples than those employed for the microarray. Total RNA from the ovary was extracted with the RNeasy Mini kit (Qiagen), and treated with DNase I using the RNase-Free DNase kit (Qiagen). An aliquot of the RNA (0.5 μg) was reverse-transcribed using 20 IU of AMV RT (Stratagene), 0.5 μM oligo(dT)_12-18 _(Invitrogen) and 1 mM dNTPs for 1.5 h at 50°C. Real-time qPCR amplifications were performed in a final volume of 20 μl with 10 μl SYBR^® ^Green qPCR master mix (Applied Biosystems), 2 μl diluted (1:5) cDNA, and 0.5 μM of each primer (see Additional file 4). The sequences were amplified in duplicate for each sample on 384-well plates using the ABI PRISM 7900HT sequence detection system (Applied Biosystems). The amplification protocol was as follows: an initial denaturation and activation step at 50°C for 2 min, and 95°C for 10 min, followed by 40 cycles of 95°C for 15 s and 63°C for 1 min. After the amplification phase, a temperature-determinating dissociation step was carried out at 95°C for 15 s, 60°C for 15 s, and 95°C for 15 s. For normalization of cDNA loading, all samples were run in parallel using 18S ribosomal protein (*18S*) as reference gene, since its expression between experimental samples did not show significant differences (data not shown). Negative control samples were also run in which the template was not added. To estimate efficiencies, a standard curve was generated for each primer pair from 10-fold serial dilutions (from 100 to 0.01 ng) of a pool of first-stranded cDNA template from all samples. Standard curves represented the cycle threshold (Ct) value as a function of the logarithm of the number of copies generated, defined arbitrarily as one copy for the most diluted standard. All calibration curves exhibited correlation coefficients higher than 0.97, and the corresponding real-time PCR efficiencies were above 99%. Values of relative expression in ovaries at different developmental stage were statistically analyzed by one-way ANOVA. The significance level was set at 0.05.

### In situ hybridization

Samples of ovaries were fixed in 4% paraformaldehyde for 16-20 h at 4°C, and subsequently dehydrated and embedded in Paraplast (Sigma). In situ hybridization on 7-μm sections was carried out with digoxigenin-alkaline phosphatase (DIG-AP) incorporated cRNA probes as previously described [109]. DIG-AP riboprobes were synthesized with T3 and T7 RNA polymerases using the DIG RNA Labeling Kit (Roche). The DIG-labeled probes were detected as previously described [105], and the resulting dark blue to purple color indicated localization of the transcripts. Sections were examined and photographed with a Leica DMLB light microscope.

## Authors' contributions

ATS performed RNA sample preparation and quality control for microarray hibridization, real-time PCR, bioinformatic analyses together with JL, and drafted the manuscript. MJA and EA led the hormonal induction experiments and morphometric data analysis, and carried out the histological studies. FC performed the in situ hybridization experiments. JC conceived and coordinated the study, participated in the design of the experiments and analyses of data, and was in charge of writing the final version of the manuscript. All authors read and approved the final manuscript.

## Supplementary Material

Additional file 1 **Microarray hybridizations**. Scatter plot of the signal intensities in the Cy3 and Cy5 channel of replica analysis of self-to-self and differential gene expression experiments (vitellogenic *vs*. previtellogenic ovaries, mature *vs*. vitellogenic ovaries, and atretic *vs*. vitellogenic/mature ovaries). In each scatter plot, the green and blue lines parallel to the black diagonal line represent the ± 3σ limits of the data from control and *Solea senegalensis *specific oligos, respectively. The histograms of the distribution of fold changes (FC) as log2(S/C) for control and *S. senegalensis *specific oligos in each experiment are shown on the right. In these panels, the green and blue curves represent the ± 3σ limits on the data from control and *S. senegalensis *specific oligos, respectively. Click here for file

Additional file 2 **Identification of clone pgsP0015C05 **(GenBank accession number FF286365)** as Senegalese sole apolipoprotein C-I (ApoC-I)**. (A) Amino acid sequence alignment of human, zebrafish and trout ApoC-I with Senegalese sole FF286365 and sea bream AAT45249 (putative ApoC-I). In the upper line, the protein family (pfam) database domain no. 04691 (ApoC-I) is shown in bold letters. In red color, the consensus (cons) sequence between pfam04691 and each of the amino acid sequences is indicated. The asterisks at the bottom indicate fully-conserved residues. (B) Fifty percent majority-rule bootstrap consensus phylogenetic tree of teleost ApoC-I reconstructed with the NJ method (1000 replications) based on a mean (uncorrected) character distance matrix. Only nodes with > 50% bootstrap support are indicated. The GenBank accession number of the amino acid sequences is indicated for each species. Scale bar indicates the number of amino acid substitution per site. Click here for file

Additional file 3 **Amino acid sequence analysis of Senegalese sole S100 calcium-binding protein**. (A) Amino acid alignment of sole S100 with S100 proteins of the A1, A10 and B subgroups from human and other teleosts. In the upper part is indicated the typical structure of mammalian S100 proteins formed by four helices (Helix I-IV) separated by a S100 EF hand calcium-binding domain, a hinge and a canonical EF hand calcium-binding domain. Conserved residues in most of the sequences are shaded in bold, identical residues are indicated by asterisks at the bottom, and conserved amino acid substitutions and substitutions with similar amino acids are indicated by double or single dots, respectively. Conserved residues in most of the members of the S100A10 subgroup are shaded in magenta. (B) Phylogenetic tree of human and teleost S100 proteins reconstructed with the NJ method (1000 replications) based on a mean (uncorrected) character distance matrix. Only sequences belonging to the A1, A10 and B subgroups are employed for the analysis. Nodes with > 60% bootstrap support are indicated. The GenBank accession number of the amino acid sequences is indicated for each species. Scale bar indicates the number of amino acid substitution per site. Click here for file

Additional file 4 **Nucleotide sequence of the primers employed for real-time quantitative RT-PCR**. The table lists the forward and reverse oligonucleotide primers employed for qPCR. Click here for file
