# Supplementary material for: New insights into molecular pathways associated with flatfish ovarian development and atresia revealed by transcriptional analysis
Source: BMC Genomics. 2009 Sep 15;10:434. doi: 10.1186/1471-2164-10-434 (PMC2751788; doi:10.1186/1471-2164-10-434)
Supplement: Additional file 1 — Microarray hybridizations. Scatter plot of the signal intensities in the Cy3 and Cy5 channel of replica analysis of self-to-self and differential gene expression experiments (vitellogenic vs. previtellogenic ovaries, mature vs. vitellogenic ovaries, and atretic vs. vitellogenic/mature ovaries). In each scatter plot, the green and blue lines parallel to the black diagonal line represent the ± 3σ limits of the data from control and Solea senegalensis specific oligos, respectively. The histograms of the distribution of fold changes (FC) as log2(S/C) for control and S. senegalensis specific oligos in each experiment are shown on the right. In these panels, the green and blue curves represent the ± 3σ limits on the data from control and S. senegalensis specific oligos, respectively. [file 1471-2164-10-434-S1.pdf]

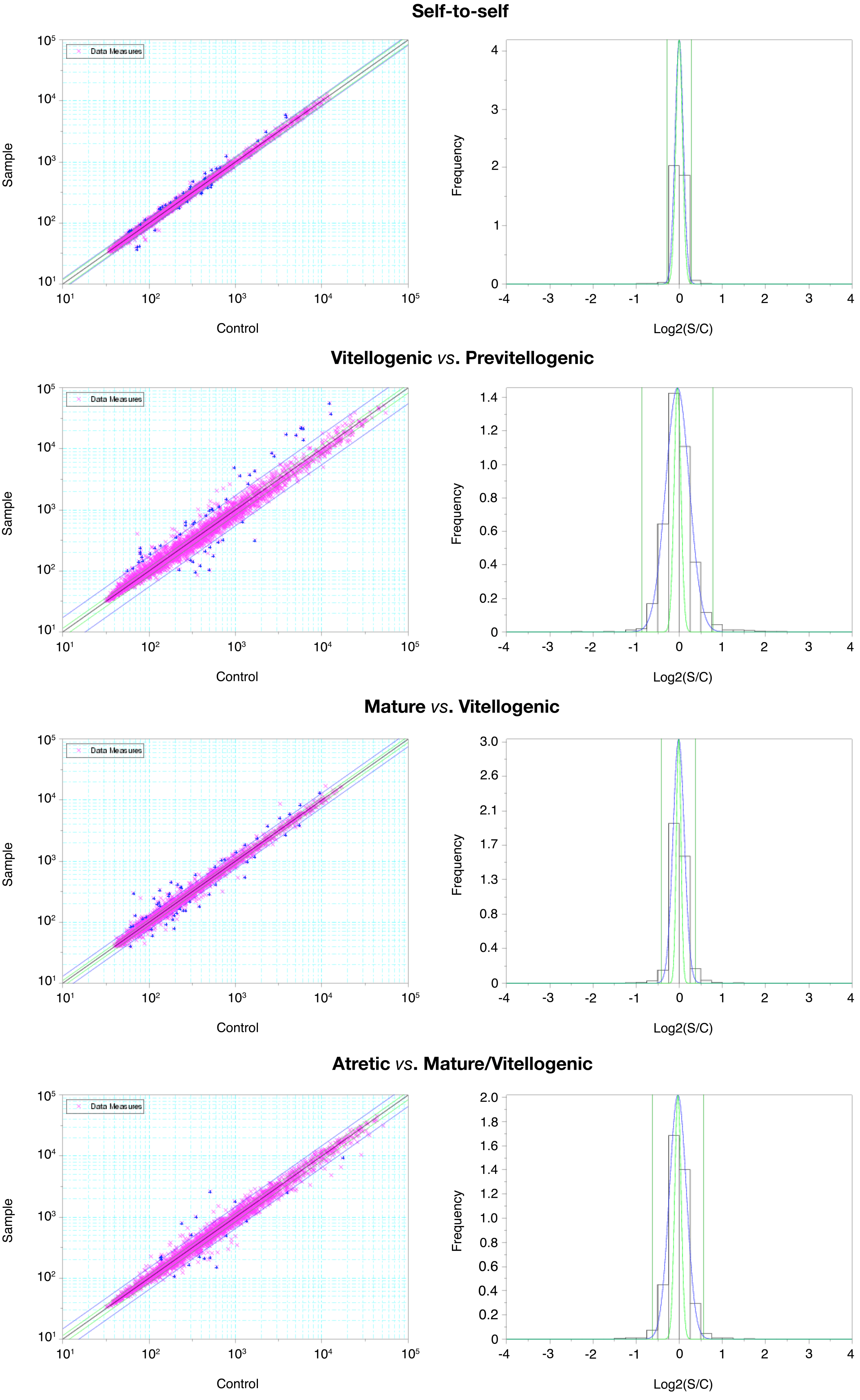
Self-to-self
Data Measures
Sample
Control
Frequency
Log2(S/C)
Vitellogenic vs. Previtellogenic
Data Measures
Sample
Control
Frequency
Log2(S/C)
Mature vs. Vitellogenic
Data Measures
Sample
Control
Frequency
Log2(S/C)
Atretic vs. Mature/Vitellogenic
Data Measures
Sample
Control
Frequency
Log2(S/C)
