# Supplementary material for: New insights into molecular pathways associated with flatfish ovarian development and atresia revealed by transcriptional analysis
Source: BMC Genomics. 2009 Sep 15;10:434. doi: 10.1186/1471-2164-10-434 (PMC2751788; doi:10.1186/1471-2164-10-434)

**A**

```
pfam04691: ApoC-1                        apdlsstlesiPDKLKEFGntLEDKAraaieriKqselpaKtRnWFsEaFkKvkeklKttf

Cons                                     +pd+ss+l    DKLKEFGntLEDKAr++i+riKqsel+aK+R+WFsE+F+KvkeklK+++
Human      1 MRLFLSLPVLVVVLSIVLEGPAPAQG-TPDVSSAL----DKLKEFGNTLEDKARELISRIKQSELSAKMREWFSETFQKVKEKLKIDS 83
Cons                                      p l   + +    ++KE     L DK  + a ++i qsel +KtRnWF + ++K k+k+  tf
Zebrafish 38 MKLYLAAAVLMLVLAVHTEA----QE-EPTLEQHFTKFGTQMKEIAEDLADKTKTAFQNIEQSELGTKTRNWFNDQYEKLKQKMTETF 121
Cons                                        +   + +   D++ E     L +KA+a +++ + se ++K +nW +E  + vk+ +
Sole       1 MRLYLAVAVLMLAFVAYTEA----Q--DDTIEERFSAFTDRVAEMSRNLAEKAKANMQEFQSSEFATKAKNWITERVEAVKNSFN--- 79
Cons                                        +   + +   +++    G  L +KA+ a ++i  se  +KtR+WF+E  +  k k++  +
Sea bream  1 MRLFLAVAVLMLAFIAYTEA----Q--EETMEQKFTQFGERVTQVGQDLAEKAKTAFDSIHNSEAMTKTRDWFAEQLEALKAKVQEIS 83
Cons                                        +   +     ++K+     L  K ++++e+i  se  +KtR WF+E F+K+k k+  tf
Trout      1 MKLSIAIAVLMLVFAAHTEA----QEAEKTIEEHFTTFGNQMKDLSEDLTVKTKDIVEKIGDSEFITKTRTWFTEQFDKMKAKIDETF 87
               *     **        *                              *  *          **   *   *        *
```

**B**

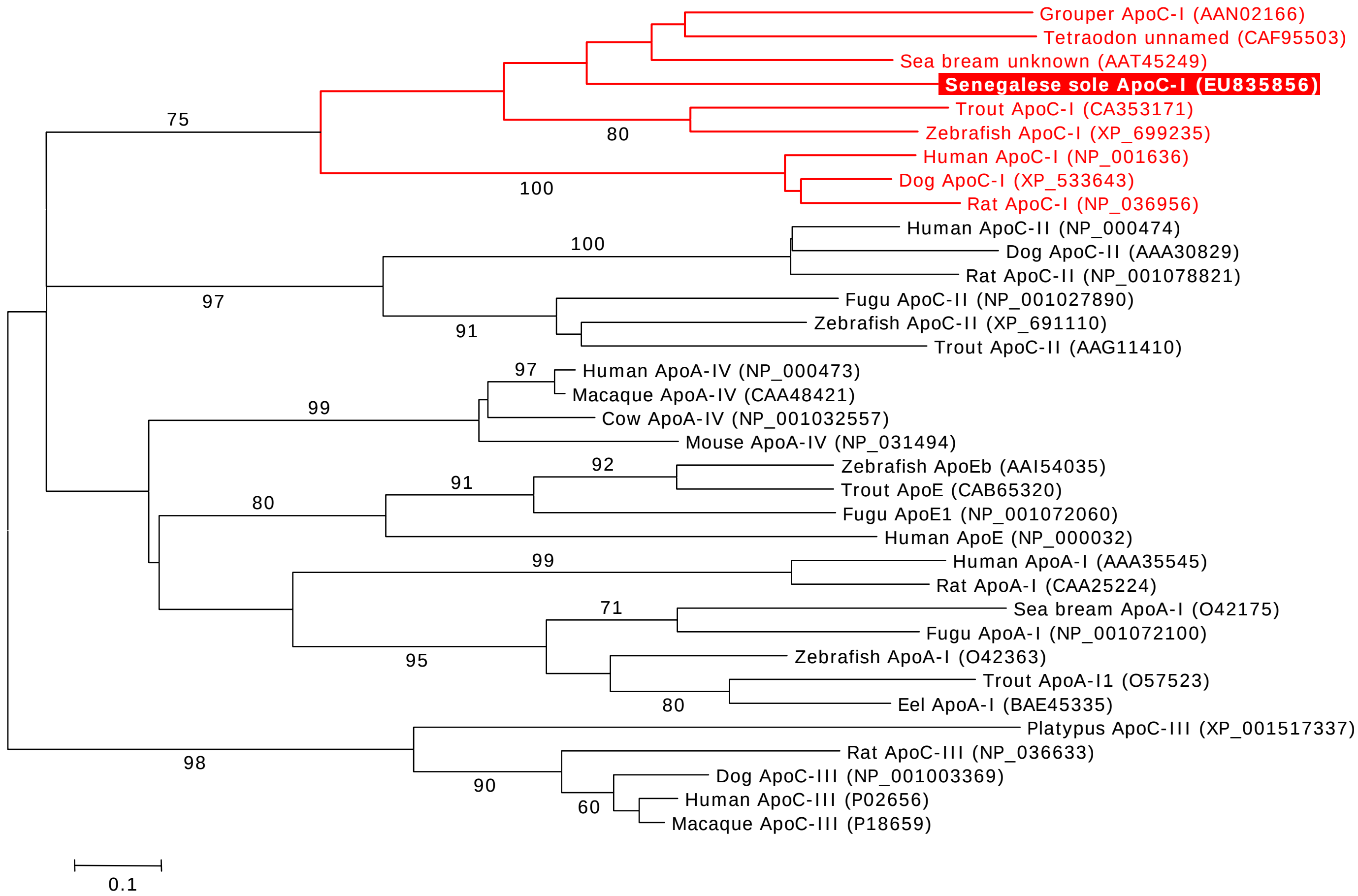

Supplement: Additional file 2 — Identification of clone pgsP0015C05 (GenBank accession number FF286365) as Senegalese sole apolipoprotein C-I (ApoC-I). (A) Amino acid sequence alignment of human, zebrafish and trout ApoC-I with Senegalese sole FF286365 and sea bream AAT45249 (putative ApoC-I). In the upper line, the protein family (pfam) database domain no. 04691 (ApoC-I) is shown in bold letters. In red color, the consensus (cons) sequence between pfam04691 and each of the amino acid sequences is indicated. The asterisks at the bottom indicate fully-conserved residues. (B) Fifty percent majority-rule bootstrap consensus phylogenetic tree of teleost ApoC-I reconstructed with the NJ method (1000 replications) based on a mean (uncorrected) character distance matrix. Only nodes with > 50% bootstrap support are indicated. The GenBank accession number of the amino acid sequences is indicated for each species. Scale bar indicates the number of amino acid substitution per site. [file 1471-2164-10-434-S2.pdf]
