# Supplementary figures and images for: New insights into molecular pathways associated with flatfish ovarian development and atresia revealed by transcriptional analysis
Source: BMC Genomics. 2009 Sep 15;10:434. doi: 10.1186/1471-2164-10-434 (PMC2751788; doi:10.1186/1471-2164-10-434)

**A**

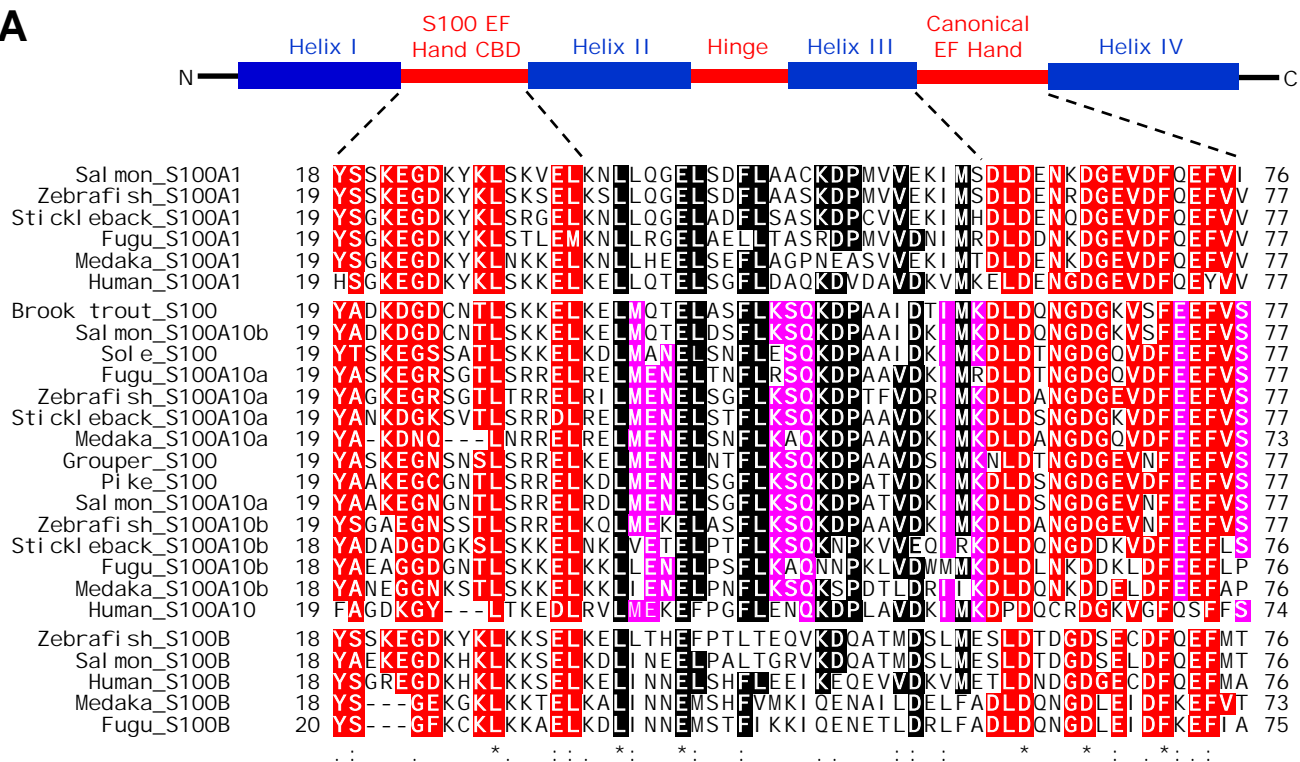

**B**

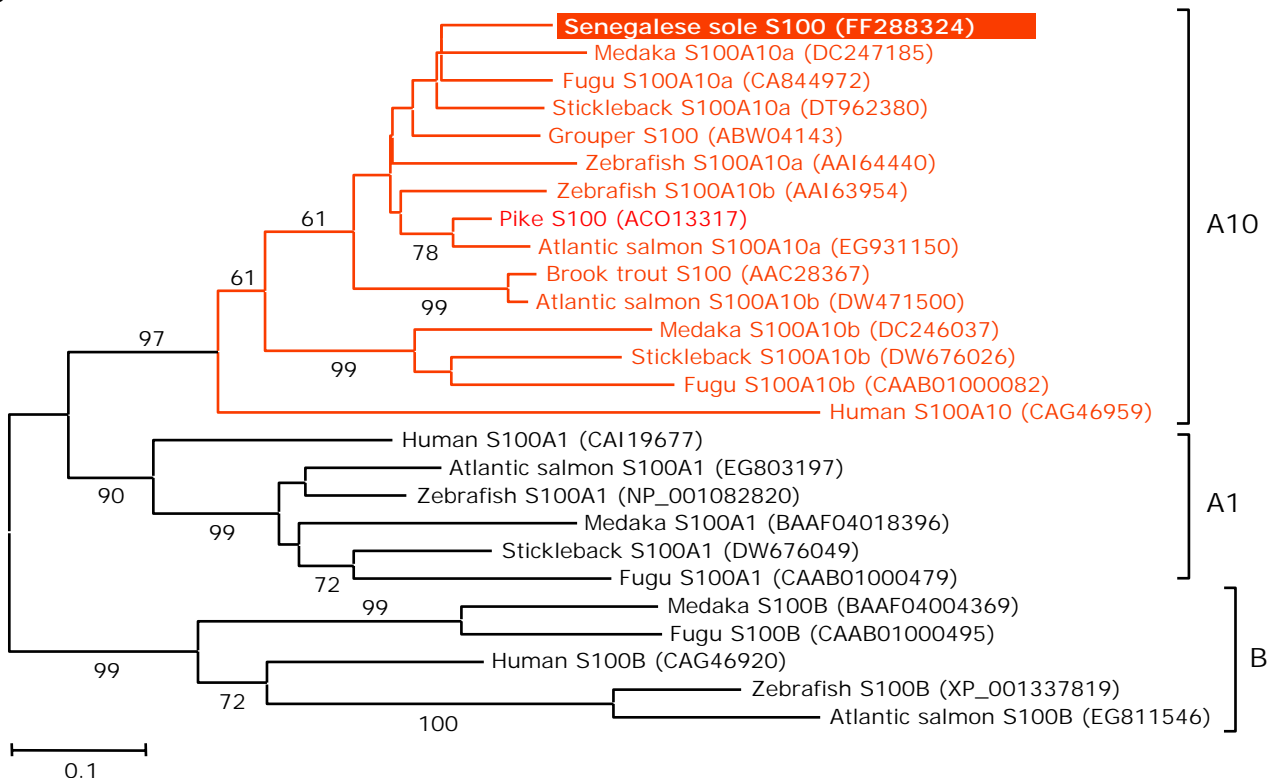

Supplement: Additional file 3 — Amino acid sequence analysis of Senegalese sole S100 calcium-binding protein. (A) Amino acid alignment of sole S100 with S100 proteins of the A1, A10 and B subgroups from human and other teleosts. In the upper part is indicated the typical structure of mammalian S100 proteins formed by four helices (Helix I-IV) separated by a S100 EF hand calcium-binding domain, a hinge and a canonical EF hand calcium-binding domain. Conserved residues in most of the sequences are shaded in bold, identical residues are indicated by asterisks at the bottom, and conserved amino acid substitutions and substitutions with similar amino acids are indicated by double or single dots, respectively. Conserved residues in most of the members of the S100A10 subgroup are shaded in magenta. (B) Phylogenetic tree of human and teleost S100 proteins reconstructed with the NJ method (1000 replications) based on a mean (uncorrected) character distance matrix. Only sequences belonging to the A1, A10 and B subgroups are employed for the analysis. Nodes with > 60% bootstrap support are indicated. The GenBank accession number of the amino acid sequences is indicated for each species. Scale bar indicates the number of amino acid substitution per site. [file 1471-2164-10-434-S3.pdf]
