# Supplementary material for: New insights into molecular pathways associated with flatfish ovarian development and atresia revealed by transcriptional analysis
Source: BMC Genomics. 2009 Sep 15;10:434. doi: 10.1186/1471-2164-10-434 (PMC2751788; doi:10.1186/1471-2164-10-434)
Supplement: Additional file 4 — Nucleotide sequence of the primers employed for real-time quantitative RT-PCR. The table lists the forward and reverse oligonucleotide primers employed for qPCR. [file 1471-2164-10-434-S4.pdf]

Nucleotide sequences of the primers employed for real-time qPCR

Clone ID	GenBank accession	Transcript symbol	Forward sequence	Reverse sequence
pgsP0002C23	FF282126	*cyfip1*	TGCAGGTGTTGATGGGTCGT	GCAGAGCTGGGTCGTTGTGA
pgsP0006E10	FF283378	*ctbp1*	ACAAAACCGCGAGTGACATC	GCCACAGAGGGTGGCTAAAC
pgsP0007K07	FF283810	*lect2l*	CTGCAGTGGAAACCCAAACA	GTCACGTCAAACGGAGCGTA
pgsP0008C17	FF283994	*apoa1*	TTGAGGCTAATCGTGCCAAA	CCTGCGTGCTTGTCCTTGTA
pgsP0010O12	FF284909	*thbs1*	CGCGTCCTCTTCTCAGACCT	GCGTCTGGTGCATCTCAGTT
pgsP0012B12	FF285326	*BRAFLDRAFT_ 128798*	GAGTTTCTGCGCAAATGCAG	GTGACACAGCGGGTGATCTT
pgsP0013B15	FF285673	*tob1a*	GCCCCCTGCAGTACAACAAT	TTTGCATGTACTAGTTGGCCATAA
pgsP0013L08	FF285880	*si:ch211-81a5.7*	TGGTGCAGGGCTCTCGTGTA	CTTGTGGCTGCAGAGGTGGA
pgsP0015C05	FF286365	*apoc1*	CGTGCTGATGCTGGCTTTTG	TTGCAAACTCGCTGCTCTGG
pgsP0015N21	FF286629	*sepw2a*	GGCCAGTGCTACTGTAAAATGC	GAAAGCAGCGAGCTGTTAGG
pgsP0016M08	FF286940	*atp1b1a*	CACCGTTACTTTGCCGTTCA	CTAGGCTGGACCCACAAAGC
pgsP0017B15	FF287055	*a2m*	AGGACATTCCCATCCACCAC	AGCCTTGTCACTTGGCTGGT
pgsP0019B22	FF287743	*hsp90b*	TGGACCAGAAACCCTGATGA	CAGCTGACCCTCCACTGAGA
pgsP0020D03	FF288118	*zp3*	TCCATGCTCCCCTTCGTGTT	CTTGAAAGCGGCCCATGAAC
pgsP0020M08	FF288324	*s100a10*	TGCCATGGAGTCCCTTATCA	TCGATAGCAGCAGGGTCCTT
pgsP0022B24	FF288799	*mkrn1*	AGCCCTGCCCCTCTCCTAC	GCACTGCCACAATACGAAGG
pgsP0022J20	FF288970	*mapk13*	ACGGGTATCGACCTGCTGGA	GTGGCGTTGTCATGGCTGTC
pgsP0023H12	FF289266	*LOC100005008*	TGGTTGTTGCTGTGGGAGGA	GACACGCTCCTGTCCCTGGT
pgsP0025P21	FF290169	*LOC100090881*	TGCAACCAATGCTGTTAGGG	ATCCTCAGCTCGCAACACCT
pgsP0027C08	FF290565	*acaa2*	GAGGCGTTTGCCCCTCAGTA	GCTCCAGAAGCAGCGAGAGG
	EF126042	*18s*	GAATTGACGGAAGGGCACCACCAG	ACTAAGAACGGCCATGCACCACCAC
